# Phosphorylated STYK1 restrains the inhibitory role of EGFR in autophagy initiation and EGFR-TKIs sensitivity

**DOI:** 10.1016/j.cellin.2022.100045

**Published:** 2022-06-16

**Authors:** Cefan Zhou, Xueying Dong, Ming Wang, Xuehong Qian, Miao Hu, Kai Liang, Yanyan Liang, Rui Zhang, Yuan Huang, Hao Lyu, Shuai Xiao, Yongfei Tang, Declan William Ali, Marek Michalak, Xing-Zhen Chen, Jingfeng Tang

**Affiliations:** a National "111" Center for Cellular Regulation and Molecular Pharmaceutics, Key Laboratory of Fermentation Engineering (Ministry of Education), Hubei University of Technology, Wuhan, China; b Membrane Protein Disease Research Group, Department of Physiology, Faculty of Medicine and Dentistry, University of Alberta, Edmonton, AB, Canada; c Department of Clinical Laboratory, Renmin Hospital of Wuhan University, Wuhan, China; d Department of Pathology, Renmin Hospital of Wuhan University, Wuhan, China; e Department of Biological Sciences, University of Alberta, Edmonton, Alberta, Canada; f Department of Biochemistry, University of Alberta, Edmonton, Alberta, Canada

**Keywords:** STYK1, EGFR, Autophagy initiation, Resistance, NSCLC

## Abstract

Epidermal growth factor receptor (EGFR) plays critical roles in cell proliferation and tumorigenesis. Autophagy has emerged as a potential mechanism involved in the acquired resistance to anti-EGFR treatments, however, the molecular mechanisms has not been fully addressed. In this study, we identified EGFR interacts with STYK1, a positive autophagy regulator, in EGFR kinase activity dependent manner. We found that EGFR phosphorylates STYK1 at Y356 site and STYK1 inhibits activated EGFR mediated Beclin1 tyrosine phosphorylation and interaction between Bcl2 and Beclin1, thus enhances PtdIns3K-C1 complex assembly and autophagy initiation. We also demonstrated that STYK1 depletion increased the sensitivity of NSCLC cells to EGFR-TKIs *in vitro* and *in vivo.* Moreover, EGFR-TKIs induced activation of AMPK phosphorylates STYK1 at S304 site. STYK1 S304 collaborated with Y356 phosphorylation to enhance the EGFR-STYK1 interaction and reverse the inhibitory effects of EGFR to autophagy flux. Collectively, these data revealed new roles and cross-talk between STYK1 and EGFR in autophagy regulation and EGFR-TKIs sensitivity in NSCLC.

## Highlights

• STYK1 binds and restrains the role of EGFR in autophagy inhibition upon EGF stimulation.
• STYK1 is phosphorylated by EGFR at Y356 and enhances the dissociation of EGFR from the PtdIns3K-C1 complex.
• EGFR-TKIs induced activation of AMPK phosphorylated STYK1 at S304 site.
• STYK1 S304 collaborated with Y356 phosphorylation to enhance autophagy initiation and NSCLC EGFR-TKIs resistance.

## Graphical abstract

**Figure:**
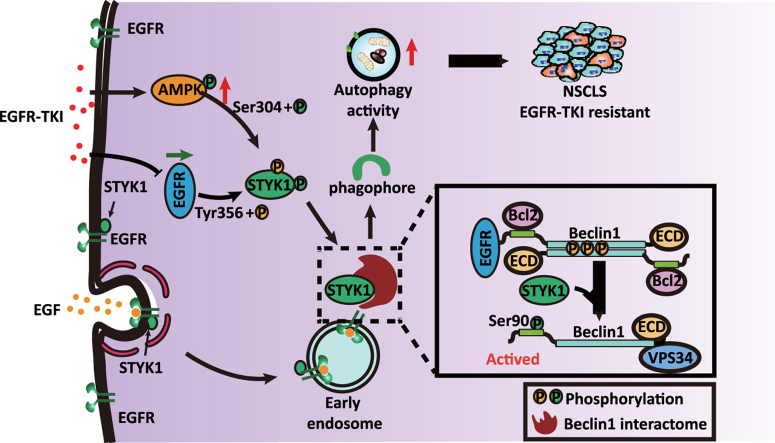

## 1 Introduction

Macroautophagy/autophagy is an evolutionarily conserved degradation process that helps to remove unfolded proteins and damaged organelles and provides the necessary nutrients for cell survival, especially under various stress conditions (Klionsky et al., 2021). Autophagy initiation requires a series of autophagy-related (ATG) proteins that function in a sequential order (Ohsumi, 2014). The PtdIns3K-C1 complex, which contains vacuolar protein sorting 34 (VPS34), vacuolar protein sorting 15 (VPS15), Beclin1 and ATG14, plays a central role in the initiation stage of autophagy and is regulated by AMPK activation or MTOR inhibition in response to nutrient depletion (Zhou et al., 2021). Several Beclin1 interacting partners, such as the anti-apoptotic protein Bcl2 (and other Bcl2 family members), Rubicon, and Dapper1, showed distinguish regulatory role in mammalian cells in response to starvation stress(Sun et al., 2011). For example, through binding to the BH3 domain of Beclin1, Bcl2 facilitating Beclin1 release from the PtdIns3K complex and in turn inhibit the activity of autophagy. Rubicon suppresses the lipid kinase activity of UVRAG-linked VPS34 complex through direct interaction with Beclin1 and UVRAG during multiple steps of the autophagic process (Zhong et al., 2009). Loss-of-function mutations of several autophagy-related genes, such as Beclin1, ATG5 and ATG7, results in spontaneous tumorigenesis (Rubinsztein et al., 2012). Moreover, autophagy stimulation during various pathological state and metabolic stress is important for the survival of eukaryotic cells (Nobili et al., 2021). Therefore, therapeutic approaches that target and intervene in autophagy are considered promising for treating pathological cellular functions, such as oncogenesis and cancer therapy resistance (Rubinsztein et al., 2012).

Epidermal growth factor receptor (EGFR) is an oncogenic receptor tyrosine kinase that links extracellular signals to the control of cell survival, growth, proliferation and differentiation (Piper-Vallillo et al., 2020). Non-small cell lung cancer (NSCLC) patients, harboring sensitizing epidermal growth factor receptor (EGFR) mutations, such as L858R point mutations and exon 19 deletions around codons 746–750, have shown significant response to EGFR tyrosine kinase inhibitors (TKIs), such as gefitinib, erlotinib and icotinib (Gonzalvez et al., 2021; Hsu et al., 2018). However, the secondary mutations in the EGFR gene (T790M) and activation of bypass pathways (such as AXL) leads to the most eventually acquired resistance among NSCLC patients that initially respond to EGFR-TKIs (Chong & Jänne, 2013; Gridelli et al., 2015). Recent studies have shown roles for EGFR signaling in autophagy suppression by activation of the PI3K/AKT/mTOR pathway (Liu et al., 2020; Wu & Zhang, 2020). Moreover, mutational activation of EGFR and ligand stimulation of wild-type EGFR has been reported to result in Beclin 1 tyrosine phosphorylation, which leads to autophagy suppression, tumor growth, and tumor dedifferentiation in NSCLC xenografts (Wei et al., 2013). Inhibition of EGFR-TKI-induced autophagy was reported to contribute to TKI resistance in NSCLC with EGFR mutations (Gorzalczany et al., 2011; Wei et al., 2013). However, the regulatory network of autophagy inhibition upon EGF stimulation patients remains obscure.

STYK1 (serine/threonine/tyrosine kinase 1), a newly identified oncogenic protein that belongs to the receptor tyrosine kinase (RTK) family, has been reported to promote many types of cancer development and metastasis through activating MEK/ERK and PI3K/AKT signaling (Huang et al., 2019; Wang et al., 2016) and facilitate the genesis and remodeling of blood and lymphatic vessels during tumor progression (Liu et al., 2016). In addition, our previous report firstly discovered that STYK1 plays an important role in modulating autophagy through improving ATG14-associated class III PI3K activity in the basal condition or in response to nutrient starvation (Zhou et al., 2020a). However, little is known about the cross-talk between STYK1 and EGFR in autophagy regulation. In this study, we investigated the potential function of STYK1 in EGFR-mediated autophagy in NSCLC cells and revealed that STYK1 inhibits the effects of EGFR to Beclin1 phosphorylation and Beclin1 interactome assembly. We also demonstrated STYK1 could be phosphorylated by EGFR upon EGF stimulation or by AMPK upon EGFR-TKI treatment.

## 2 Materials and methods

### 2.1 Cell lines, reagents, and antibodies

Human non small cell lung cancer cell line A549 and HCC827 were purchased from the National Collection of Authenticated Cell Cultures, Chinese Academy of Sciences (SCSP-538 and SCSP-503). HEK293T and HeLa cells were stored in our lab. ATG7 knockout MEF cell line was a gift from prof. Xuejie Yu (Wuhan University). Gefitinib and erlotinib were purchased from MedChemExpress (HY-50895 and HY-50896). Autophagy inhibitor chloroquine diphosphate salt (CQ, 10 μM) was purchased from SangonBiotech (Shanghai, A506569). AMPK inhibitor compound C and activator AICAR were purchased from Selleck (S7306 and S1802). EBSS (Hyclone, SH30024.01) starvation in this study was carried out to switch the culture medium from the complete medium to the EBSS medium unless described otherwise. Commercially available antibodies and dilutions used are as follows: anti-EGFR (Proteintech, 18986-1-AP; 1:1000 dilution), anti-STYK1 (Abcam, ab97451; 1:1000 dilution), anti-mouse HA (EMD Millipore, M180-3; 1:2000 dilution), anti-Rabbit HA (Proteintech, 51064-2-AP; 1:2000 dilution), anti-mouse DYKDDDDK (MBL, M185; 1:2000 dilution), anti-Rabbit DYKDDDDK (Proteintech, 80010-1-RR; 1:2000 dilution), anti-GAPDH (Proteintech, 60004-1-Ig; 1:5000 dilution), anti-p-EGFR Y1068 (Cell Signaling Technology, 3777; 1:1000 dilution), anti-p-ERK T202/Y204 (Cell Signaling Technology, 4370; 1:1000 dilution), anti-ERK1/2 (Cell Signaling Technology, 4695; 1:1000 dilution), anti-p-AKT S473 (Cell Signaling Technology, 4058; 1:1000 dilution), anti-AKT (Proteintech, 10176-2-AP; 1:1000 dilution), anti-LC3 (Cell Signaling Technology, 4108; 1:1000 dilution), anti-PIK3C3/VPS34 (Proteintech, 10828-1-AP; 1:1000 dilution), anti-Beclin1 (Proteintech, 11306-1-AP; 1:1000 dilution), anti- PIK3R4/VPS15 (Santa Cruz Biotechnology, sc-100798; 1:500 dilution), anti-Rubicon (Proteintech, 21444-1-AP; 1:1000 dilution), anti-GFP (EMD Millipore, 598; 1:1000 dilution), anti-Bcl2 (Proteintech, 12789-1-AP; 1:1000 dilution), anti-phosphotyrosine (Sigma, P4110; 1:1000 dilution), anti-p-AMPKα2-T172 (Cell Signaling Technology, 2535; 1:1000 dilution), anti-AMPKα2 (Proteintech, 18167-1-AP; 1:1000 dilution), anti-ATG14/Barkor (Proteintech, 19491-1-AP; 1:1000 dilution), anti-phosphoserine (EMD Millipore, 05-1000x; 1:1000 dilution), anti-phospho-threonine (Cell Signaling Technology, 9386S; 1:1000 dilution), anti-ATG7 (Proteintech, 10088-2-AP; 1:1000 dilution), anti-AMPK substrate (Cell Signaling Technology, cat: 5759, 1:1000 dilution).

### 2.2 Plasmids, cell culture, siRNAs and transfection

ptfLC3 (Mammalian expression of rat LC3 fused to mRFP and GFP) was a gift from Tamotsu Yoshimori (Addgene, 21074). A549, HCC827, HEK293T and HeLa cells were cultured in Dulbecco's modified Eagle's medium (DMEM; Hyclone, SH30022.01). All culture mediums were supplemented with 10% fetal bovine serum (Biological Industries, 04-001-1ACS), 100 U/ml penicillin G and 100 μg/ml (Biosharp, BL505A) streptomycin at 37 °C in a humidified incubator containing 5% CO2. The medium was replaced every 2–3 days and the cell was subcultured and used for an experiment at 80–90% confluence. DNA and interference RNA were transfected with Lipofectamine 3000 (Invitrogen, L3000015) according to the manufacturer's instructions. shRNA for STYK1 #1 was 5ʹ- CCAUCUUUCGAGCCAAUAUTT -3ʹ. #2 was 5ʹ- GGAUGGUCUUCUCUAUGAUTT -3ʹ. siRNA for EGFR #1 was 5ʹ- CGCAAAGUGUGUAACGGAAUA -3ʹ, siRNA for EGFR #2 was 5ʹ- GCAAAGUGUGUAACGGAAUAGGUAU -3ʹ.

### 2.3 Lentiviral production and creation of stable cell lines

Lentiviral production was performed as described previously (Zhou et al., 2020b). Briefly, STYK1 shRNAs or scramble RNA were subcloned into the lentiviral vector pLKO.1-puro (Sigma, 8453). For the lentiviral particle production, 5 μg pLKO.1-STYK1 shRNA vector were co-transfected with viral packaging plasmids 3 μg psPAX2 and 3 μg pMD2.G into 293T cells in 10 cm dishes. The viral supernatant was harvested at 48 h post-transfection and filtered through a 0.22 μm membrane. After applying the viral supernatant to HCC827 cells with 10 μg/μl of polybrene (Solarbio, H8761), selection for puromycin (Solarbio, IP1280) resistance was initiated 48 h after transfection. The selection medium was changed every 2–3 d for several weeks, and clones of puromycin resistant cells were isolated and expanded for further characterization.

### 2.4 Immunoprecipitation and western blotting

Immunoprecipitation and western blotting were performed as described previously (Zhou et al., 2021). Briefly, cells were washed twice and then lysed with RIPA lysis buffer (50 mM Tris-HCl pH 7.4, 150 mM NaCl, 1% Triton X-100 [Sangon Biotech, 9002-93-1], 10 mM NaF, and 1 mM EDTA) containing proteinase inhibitor cocktail (Biomake, B14001) and Halt phosphatase inhibitor cocktail (Thermo Fisher Scientific, 78420). Protein concentration was measured using a BioRad Protein Assay kit (BioRad, 5000006). Cell lysates were incubated overnight with primary antibodies after pretreatment with IgG and protein A/G magic beads (Biomake, B23202), and then incubated with protein A/G magic beads for 2 h at 4 °C. The immunoprecipitate or cell lysates was boiled in 2 × SDS loading buffer (100 mM Tris-HCl, pH 6.8, 4% [wt:vol] SDS, 200 mM dithiothreitol, 0.2% [wt:vol] bromophenol blue, and 20% [vol:vol] glycerol [Biosharp, BS154]) for 8 min at 98 °C and then loaded on SDS-PAGE gels. The gels were then blotted onto a 0.45-μm polyvinylidene fluoride membrane (PVDF; Millipore, IPFL85R). After incubated with primary antibodies and secondary antibodies, the signals of the protein in the PVDF membranes were detected using Supersignal West Pico Plus (Invitrogen, 34580) according to the manufacturer's instructions.

### 2.5 Cell viability assay

Cell viability was determined by MTT assay performed as described previously (Zhou et al., 2016). Briefly, HCC827 cells with STYK1 knocking down with or without gefitinib or erlotinib treatment were stained with 100 μl of sterile MTT dye (0.5 mg/ml; Sigma, M2128) for 4 h at 37 °C, and then added 150 μl of DMSO (Sigma, W387520). The number of viable cells was assessed by measurement of the absorbance at 450 nm with a microplate reader. All experiments were performed in triplicate.

### 2.6 Colony formation assay

Colony formation assay was performed as described previously (Huang et al., 2022). HCC827 cells with STYK1 knocking down with or without gefitinib or erlotinib treatment were fixed with 4% paraformaldehyde and stained with 2% crystal violet. Images were obtained and the number of colonies was counted.

### 2.7 Immunofluorescence and confocal microscopy

HeLa or HCC827 cells transfected with GFP-LC3, GFP-ATG14, GFP-WIPI1, GFP-ZFYVE1 and another co-transfected overexpression or knockdown plasmids were grown on 12-well plates, and cells cultured for 48 h at 60% density were used for confocal microscopy on glass chambers. After the cells were fixed and stained with DAPI (Solarbio, C0065), images were obtained with a confocal laser-scanning microscope (Leica SP8, Wetzlar, Germany) using a 63 × oil immersion objective. Data analysis was performed using Leica LAS AF Lite software. The numbers of GFP-LC3, GFP-ATG14 and GFP-ZFYVE1 puncta per cell were assessed in 10 non-overlapping fields.

### 2.8 TUNEL assay

Apoptotic cells among HCC827 cells with STYK1 knocking down with or without gefitinib or erlotinib treatment were examined with the One Step TUNEL (TdT-mediated dUTP nick-end labeling) Apoptosis Assay Kit (Beyotime, C1090) following the manufacturer's protocols. Cells were photographed under an Olympus FSX100 microscope (Tokyo, Japan).

### 2.9 5-Ethynyl-20-deoxyuridine (EdU) incorporation assay

The EdU assay was performed as described previously (Zhou et al., 2020b). EdU labeled HCC827 cells with STYK1 knocking down with or without gefitinib or erlotinib treatment were examined with the BeyoClick™ EdU Cell Proliferation Kit with Alexa Fluor 555 (Beyotime, C0075S). Cells were photographed under an Olympus FSX100 microscope.

### 2.10 Transmission electron microscopy (TEM)

Transmission electron microscopy was performed as described previously (Zhou et al., 2019). HeLa cells were fixed in 2.5% glutaraldehyde without washing at 37 °C and further fixed with 2% osmium tetroxide buffer. Then, the fixed cells were dehydrated using a graded ethanol series and embedded in Epon. Electron microscopy was performed with a JEM-100CXII TEM transmission electron microscope (Joel, Tokyo, Japan).

### 2.11 Subcutaneous xenograft experiments

Subcutaneous xenograft experiments were performed as described previously (Zhou et al., 2020b). The subcutaneous xenograft mouse model was used to assess the tumor formation ability of HCC827 cells stably knockdown STYK1 treated with or without gefitinib. All animal experiments were carried out in compliance with a protocol specifically approved for the use of laboratory animals by the Hubei University of Technology Animal Care and Use Committee. Four-week-old (18–22 g) female BALB/c nude mice were purchased from Vital River Laboratory Animal Technology (Beijing, China). cells (3 × 10^6^) were subcutaneously injected into the left and right axillae of 3 female BALB/c nude mice per group. Two weeks later, the animals were treated with gefitinib at 50 mg/kg body weight via intraperitoneal injection twice a week. The length and width of the mouse tumors were measured every 5 days with calipers, and tumor volume (V) was calculated with the following formula: V = [(length × width × 2)/2]. Experimental mice were euthanized at the end of the observation period, and then the tumors were excised and weighed.

### 2.12 PIK3C3/VPS34 kinase activity assay

PIK3C3/VPS34 kinase activity assay was performed as described previously (Zhou et al., 2020a). Briefly, PIK3C3 was immunoprecipitated using an anti-ATG14 antibody after co-transfection with EGFR L858R/Δ746-750 mutant and MYC-STYK1 in the condition of starvation or gefitinib treatment. Then the immunoprecipitates were incubated with kinase reaction buffer (100 mM HEPES [ThermoFisher, 15,630,080] [pH 7.5], 300 mM NaCl, 2 mM CHAPS [ThermoFisher, 28,300], 10 mM MnCl2, 2 mM DTT, 100 μM ATP [Sangon Biotech, A600020]) and phosphatidylinositol substrate (Echelon, K-3000) and incubated at room temperature for 2 h. The concentration of PtdIns3P in the reaction mixture was calculated according to the manufacturer's instructions (Echelon, K-3000).

### 2.13 Immunohistochemistry

Immunohistochemistry was performed as described previously (Zhou et al., 2021). Briefly, antibodies against Ki67, LC3 and p62 were tested on sections from excised xenograft tumor tissues.

### 2.14 Statistical analysis

All experiments were performed independently at least three times. All statistical analysis were performed using GraphPad Prism 6.0 software (GraphPad, La Jolla, CA, USA). All data are presented as the mean ​± ​SD (standard deviation) from triplicates. Differences with a p value ​< ​0.05 were statistically significant. Differences between two groups were analyzed by independent sample t-tests and differences among multiple groups by one-way ANOVA; ∗represents P ​< ​0.05, ∗∗represents P ​< ​0.01 and ∗∗∗represents P ​< ​0.001.

## 3 Results

### 3.1 STYK1 interacts with EGFR and amplifies EGFR signaling

**Fig. 1:**
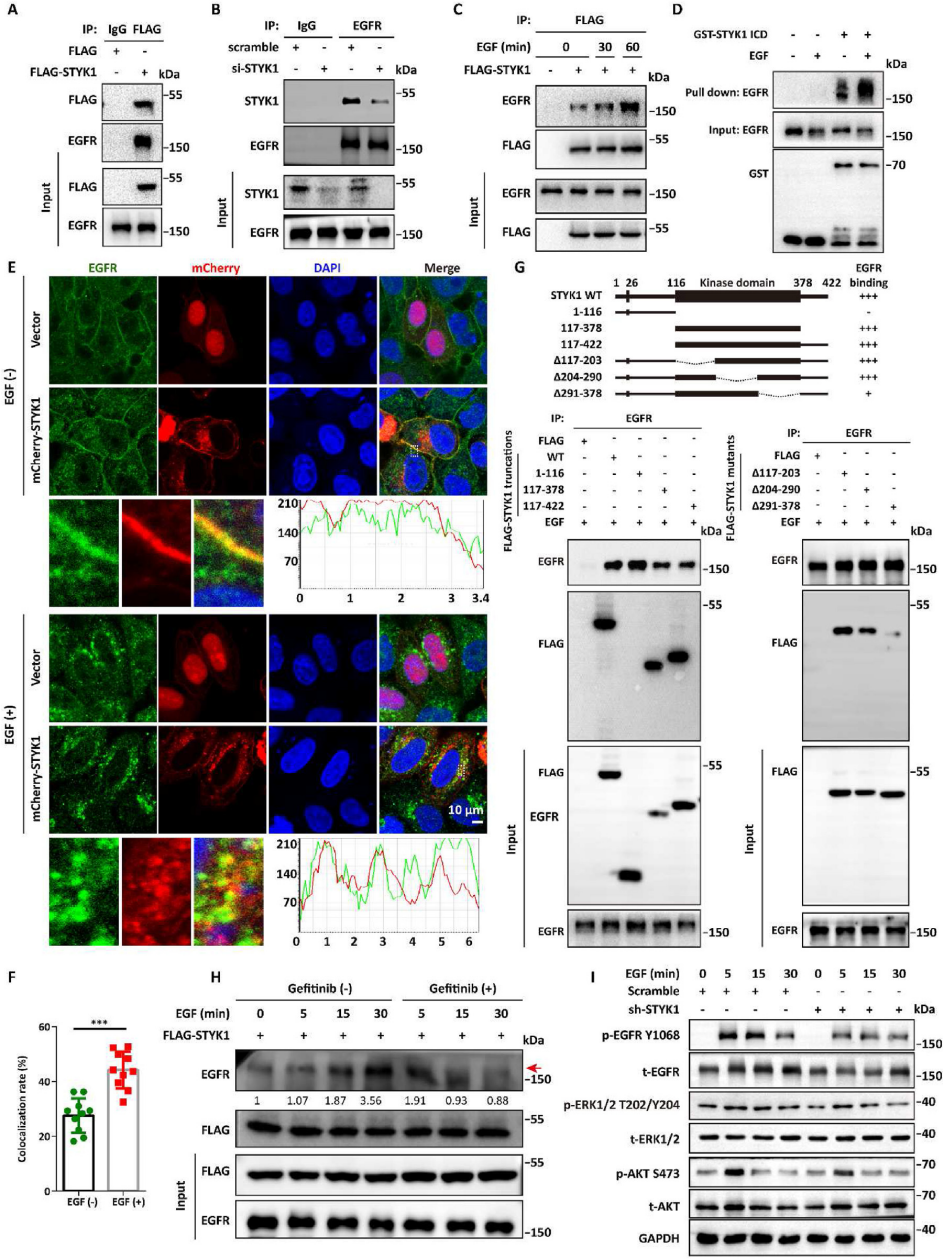
**STYK1 interacts with EGFR and amplifies EGFR signaling.(A)** HEK293T cells were transfected with FLAG-STYK1 plasmid for 48 h, then the cell lysates were used for co-IP assay using FLAG antibody and subsequent western blotting assay. (**B**) The cell lysates of HeLa cells with or without STYK1 knockdown were used for co-IP assay using EGFR antibody and subsequent western blotting assay. (**C**) The interaction between FLAG-STYK1 and EGFR in HeLa cells treated with EGF (100 ng/mL) for indicated times. (**D**) The purified GST-tagged N-terminal ectodomain (1–26 aa) and transmembrane domain (27–46 aa)-truncated STYK1 (STYK1 ICD) was incubated with the lysates from HeLa cells treated with EGF and subjected for western blotting assay. (**E and F**) The co-localization between endogenous EGFR and mCherry-STYK1 in HeLa cells after treatment with or without EGF. (**G**) Schematic of STYK1 truncation and the interaction between STYK1 and EGFR from co-IP assays in HEK293T cells transfected with indicated STYK1 truncation in the condition of EGF treatment. (**H**) Interaction between FLAG-STYK1 and EGFR in HCC827 cells treated with or without EGF and/or gefitinib (1 μM). (**I**) Western blotting analysis were used to analyze the protein expression levels of p-EGFR Y1068, p-ERK1/2 T202/Y204 and p-AKT S473 in A549 ​cells stably knockdown STYK1 upon treatment with EGF for indicated times. Data were represented as mean ​± ​SD, ∗P ​< ​0.05; ∗∗P ​< ​0.01; ∗∗∗P ​< ​0.001.

In our previous interactome screening of STYK1 using immunoprecipitation combined with mass spectrometry assay (IP-Mass Spec) (Zhou et al., 2020a), we found peptides of EGFR were detected as one of the top hits of STYK1 immunoprecipitates. To verify the interaction of EGFR and STYK1, co-immunoprecipitation (co-IP) assay using specific FLAG antibody was carried out and the results showed that EGFR was exactly existed in the immunoprecipitates of STYK1 (Fig. 1A). By using the antibody of EGFR as a bait, we also confirmed that EGFR interacted with STYK1, and the interaction was reduced while STYK1 knocked down (Fig. 1B). We also found that STYK1-EGFR interaction was significantly improved upon EGF stimulated in a time-dependent manner (Fig. 1C, Fig. S1A). By using GST-tagged N-terminal ectodomain (1–26 aa) and transmembrane domain (27–46 aa)-truncated STYK1, which we called STYK1 ICD, we confirmed that purified GST-STYK1 ICD could pull down much more EGFR from the lysates of Hela cells treated with EGF compared with cells under normal conditions (Fig. 1D). Moreover, we found that STYK1 and EGFR were colocalized in the cell membrane and shifted into cell cytoplasm upon EGF treatment accompanied with increased colocalization rate (Fig. 1E and F). To map the interaction region of STYK1 that interacts with EGFR, Four STYK1 truncation mutants were constructed and subjected to co-IP assays in HEK293T cells. The results showed that the STYK1 kinase domain (aa 117–378) mediated the interaction with EGFR (Fig. 1G). Using further deletion mutants within the kinase domain for further co-IP experiments, we found that the interaction between STYK1 and EGFR was almost abolished while the deletion of aa 291–378 compared with the other two truncations (Fig. 1G). Furthermore, we also found that the EGFR-STYK1 interaction was significantly decreased in HCC827 cells, which contains activated EGFR characterized by the exon 19 deletions around codons 746–750, in the condition of EGFR tyrosine kinase inhibitor (EGFR-TKI) gefitinib treatment (Fig. 1H). These results indicated that EGFR is a binding partner of STYK1 and the interaction is enhanced upon EGFR activation.

To investigate the role of STYK1 in EGFR signaling, gene set enrichment analysis (GSEA) was generated using the Cancer Genome Atlas (TCGA) database of lung cancer, which is characterized by hyperexpression and/or gain-of-function mutations of EGFR (Tebbutt et al., 2013), to analyze differences in the genes that are enriched in patients with high and low levels of STYK1. The results indicate that many genes that involved in EGFR signaling were enriched in the group that expressing high levels of STYK1 (Fig. S1B). Moreover, non-small cell lung cancer A549 cells that stably overexpressing STYK1 shRNA were generated and the phosphorylation levels of EGFR downstream effector were tested. The results revealed that STYK1 knockdown significantly inhibited EGF-induced phosphorylation of EGFR Y1068, ERK1/2 and AKT S473 (Fig. 1I, Fig. S1C). These data indicated that STYK1 promotes EGFR signaling activity.

### 3.2 STYK1 abrogates EGF-mediated autophagy inhibition

**Fig. 2:**
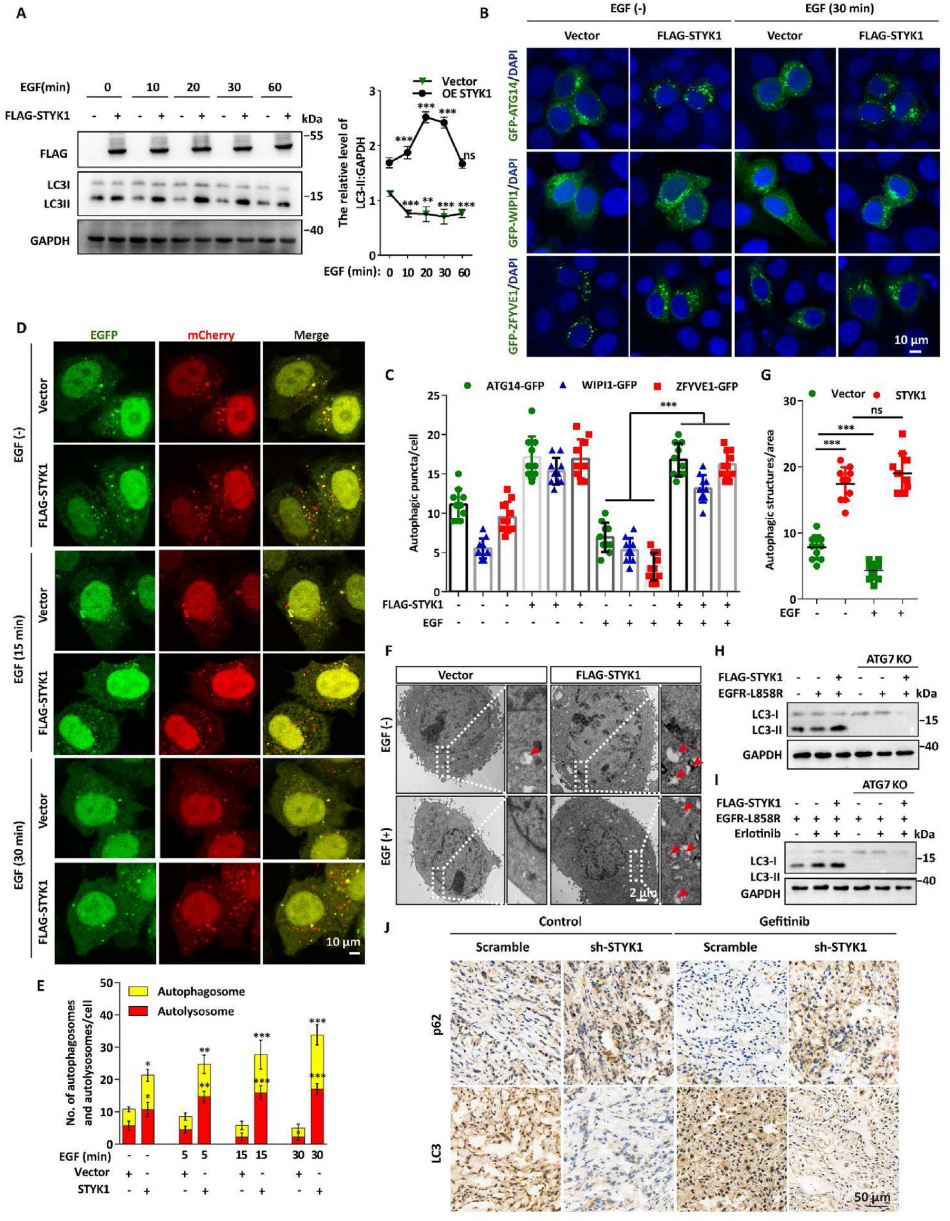
**STYK1 abrogatesEGF-mediatedautophagy inhibition.** (**A**) Western blotting analysis were used to analyze the protein expression levels of LC3-II in A549 cells overexpressing FLAG-STYK1 with or without EGF treatment. (**B**) Representative confocal images of GFP-ATG14, GFP-WIPI1 and GFP-ZFYVE1 distribution in FLAG-STYK1 transfected HeLa cells with or without EGF treatment. (**C**) The numbers of GFP-ATG14, GFP-WIPI1 and GFP-ZFYVE1 puncta were quantified (n = 10). Scale bars, 10 μm. (**D and E**) Representative confocal microscopy images of the red-only puncta and the yellow puncta in A549 cells after co-transfection with FLAG-STYK1 with or without EGF treatment for indicated times. The numbers of the autolysosomes (GFP-negative/RFP-positive, red puncta) and autophagosomes (GFP-positive/RFP-positive, yellow puncta) were quantified (n = 10). Scale bars, 10 μm. (**F and G**) Representative images of autophagosomes or autolysosomes of the A549 cells transfected with FLAG-STYK1 with or without EGF treated conditions. Both low- and high-power (zoom) images are displayed. Red arrows indicate autophagic structures. The puncta number of autophagic structures per area were quantified (n = 10). (**H and I**) Western blotting analysis were used to analyze the protein expression levels of LC3-II in MEF cells co-overexpressing FLAG-STYK1 and EGFR-L858R mutant with or without erlotinib (1 μM) treatment. (**J**) Representative immunohistochemical images of p62 and LC3 in excised HCC827 tumor tissues. Data were represented as mean ​± ​SD, ∗P ​< ​0.05; ∗∗P ​< ​0.01; ∗∗∗P ​< ​0.001.

EGFR activation was reported to inhibits autophagy (Wei et al., 2013). We then asked whether STYK1 affects EGF-mediated autophagy inhibition. To this end, we first examined the levels of LC3-II, a commonly used autophagic flux markers for autophagosome formation, after STYK1 overexpression upon EGF treatment. As expected, EGF stimulation reduced the level of LC3-II, however, STYK1 expression significantly reversed the efforts of EGF mediated LC3-II decrease (Fig. 2A; Fig. S2A). Moreover, STYK1 overexpression increased the formation of autophagic puncta after EGF treatment, as indicated by GFP-ATG14, GFP-WIPI1 and GFP-ZFYVE1, which are recruited to the phagophore assembly site and contribute to early stage autophagosome formation (Fig. 2B and C). In contrast, STYK1 knockdown markedly decreased the formation of GFP-LC3 puncta under the EGF treatment condition (Fig. S2B). We further detected the effects of STYK1 on the autophagic flux with the mRFP-GFP-LC3 tandem reporter, which enables observations of the differences between autophagosomes (GFP-positive/RFP-positive, yellow puncta) and autolysosomes (GFP-negative/RFP-positive, red puncta) after EGF treatment. The results revealed that STYK1 overexpression significantly elevated the number of autophagosomes and autolysosomes under EGF treated conditions, which also showing reversed effects on EGF-induced autophagosome generation (Fig. 2D and E). Furthermore, we also observed significantly elevated numbers of autophagic vacuoles in STYK1 overexpressed HeLa cells through transmission electron microscopy observations (Fig. 2F and G) with or without EGF treatment.

To examined whether the regulation of autophagy mediated by EGFR or STYK1 depends on the canonical autophagy machinery, ATG7 knockout (KO) MEF cells (Fig. S2C) was used and co-transfected with EGFR continuously active form (EGFR-L858R) and STYK1, treated with or without erlotinib. We found that both the effects of EGFR and STYK1 on autophagy were completed abolished in ATG7 KO cells (Fig. 2H and I). Moreover, LC3-II levels were increased after STYK1 overexpression compared with that in EGFR-L858R transfected MEF cells treated with or without erlotinib treatment (Fig. 2H and I). Additionally, we found decreased p62, and increased LC3 protein immunohistochemical staining in the extracted HCC827 xenograft tumors with STYK1 stably knockdown treated with or without gefitinib (Fig. 2J). These results indicated that STYK1 abrogates EGF-mediated canonical autophagy inhibition.

### 3.3 STYK1 inhibits EGFR-Beclin1 interaction and reverses its efforts to the assembly of Beclin1 interactome

**Fig. 3:**
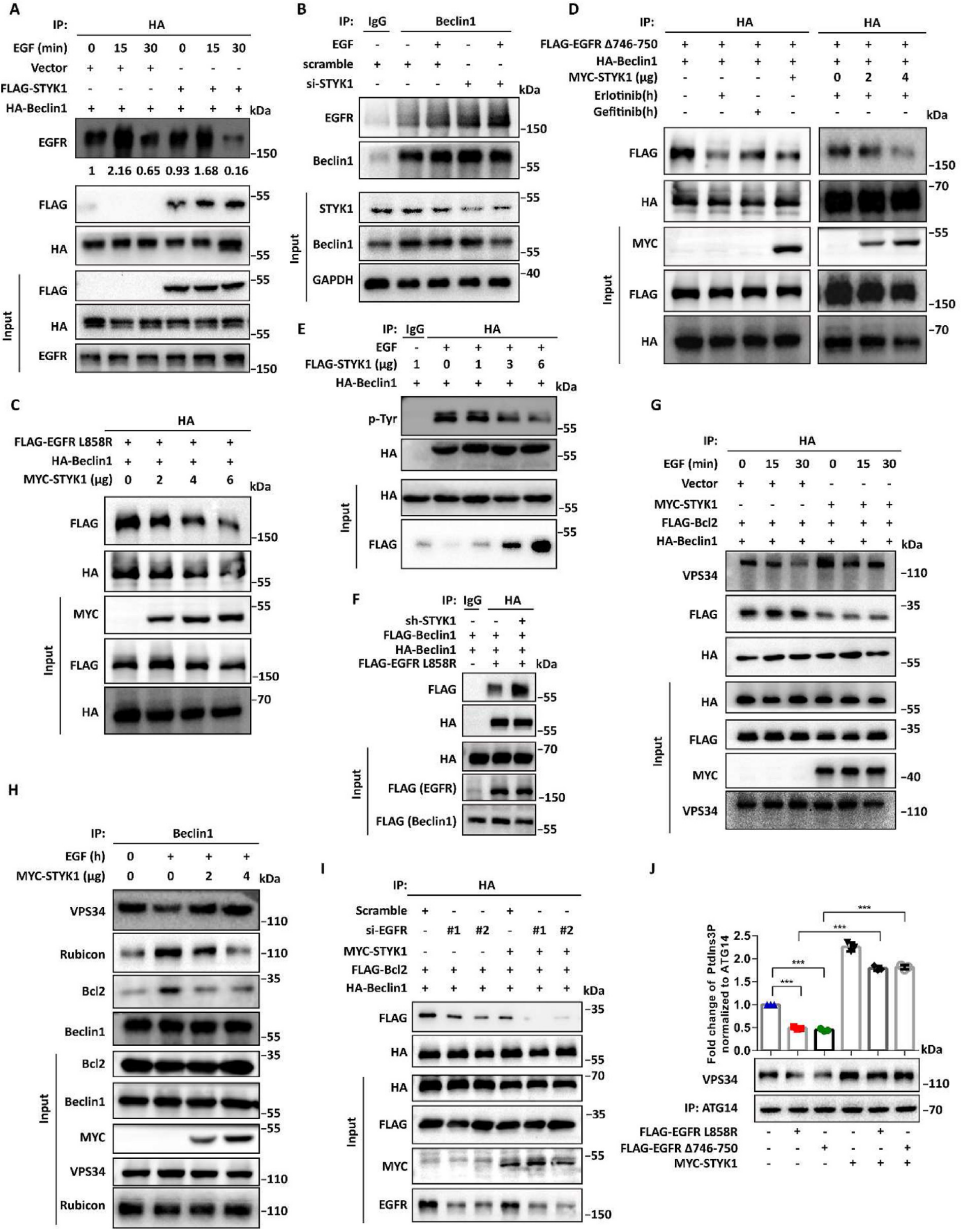
**STYK1 inhibitsEGFR-Beclin1interaction and reverses its efforts to the assembly of Beclin1 interactome.** (**A**) Interaction between HA-Beclin1 and EGFR in HEK293T cells treated with or without EGF. Cell lysates were used for IP and western blotting with indicated antibodies. (**B**) Interaction between endogenous Beclin1 and EGFR after STYK1 knockdown in HEK293T cells treated with or without EGF. Cell lysates were used for IP and western blotting with the indicated antibodies. (**C and D**) Interaction between activated EGFR L858R/Δ746-750 and HA-Beclin1 after STYK1 overexpression in HEK293T cells treated with or without EGF, erlotinib (1 μM) or gefitinib (1 μM). Cell lysates were used for IP and western blotting with the indicated antibodies. (**E**) Level of total tyrosine phosphorylation of HA-Beclin1 after STYK1 overexpression in HEK293T cells with EGF treatment. Cell lysates were used for IP and western blotting with the indicated antibodies. (**F**) Level of Beclin1 dimerization after STYK1 knockdown in EGFR L858R transfected HEK293T cells. Cell lysates were used for IP and western blotting with the indicated antibodies. (**G and H**) Interaction between Beclin1 and Bcl2, Rubicon and VPS34 after STYK1 overexpression in HEK293T cells treated with or without EGF. Cell lysates were used for IP and western blotting with the indicated antibodies. (**I**) Interaction between HA-Beclin1 and FLAG-Bcl2 after STYK1 overexpression in HCC827 cells transfected with or without EGFR siRNAs. Cell lysates were used for IP and western blotting with the indicated antibodies. (**J**) Different PtdIns3K-C1 complex components from the control or STYK1 overexpressed HEK293T cells co-transfected with EGFR L858R or Δ746-750 mutant were immunoprecipitated by the ATG14 antibody. PIK3C3 activity was measured by analyzing PtdIns3P production using the ELISA assay described in the Materials and Methods. The PtdIns3P fold change was calculated based on the concentration of PtdIns3P and was normalized to the amount of ATG14 used in the assay. Data were represented as mean ​± ​SD, ∗P ​< ​0.05; ∗∗P ​< ​0.01; ∗∗∗P ​< ​0.001.

To investigate the role of STYK1 in EGFR mediated autophagy inhibition, we firstly analyzed the alteration of EGFR-Beclin1 interaction after STYK1 overexpression/knockdown in the treatment of EGF, or co-transfected with the activated form of EGFR (EGFR Δ746-750 or EGFR-L858R) in HEK293T cells. As expected, STYK1 overexpression significantly decreased the binding ability of EGFR to Beclin1 after EGF treatment (Fig. 3A). In contrast, STYK1 knockdown elevated the interaction of EGFR and Beclin1 with or without EGF treated conditions (Fig. 3B). Moreover, we found that STYK1 does-dependently reduced the binding of EGFR-L858R with Beclin1 (Fig. 3C). Furthermore, similar to previously research (Cao et al., 2020), erlotinib and gefitinib disrupted the interaction of EGFR Δ746-750 with Beclin1, and STYK1 overexpression aggravated the effect of erlotinib on the interaction of Beclin1 with EGFR Δ746-750 (Fig. 3D).

We next investigated the role of STYK1 on EGFR mediated Beclin1 phosphorylation, and the results indicated that STYK1 reduced EGF-induced Beclin1 tyrosine phosphorylation in a does-dependent manner in A549 cells (Fig. 3E). Moreover, STYK1 decreased Beclin1 tyrosine phosphorylation after gefitinib treatment in HCC827 cells (Fig. S3). We also found that STYK1 knockdown significantly elevated the homodimerization of Beclin1 (Fig. 3F). Furthermore, we found that EGF treatment increased the interaction of Beclin1 with autophagy-inhibitory protein Rubicon and Bcl2, however, STYK1 overexpression abrogated these effects (Fig. 3G–I). STYK1 enhanced the interaction between Beclin1 and VPS34 under both EGF treated or untreated conditions (Fig. 3G and H). PIK3C3 kinase activity assays were performed to determine whether STYK1 directly modulates EGF-mediated inhibition of the activity of PtdIns3K-C1 complex. The results revealed that ATG14-linked PtdIns3K kinase activity was dramatically decreased in the cells that transfected with EGFR Δ746-750 or EGFR-L858R mutant, but significantly increased after STYK1 overexpression (Fig. 3J). These results indicated that STYK1 regulates EGF-induced autophagy inhibition through blocking the interaction EGFR with Beclin1 and its tyrosine phosphorylation, leading to alteration of the assembly of Beclin1 interactome.

### 3.4 STYK1 is phosphorylated by EGFR at Y356, which is essential for the STYK1-EGFR binding

**Fig. 4:**
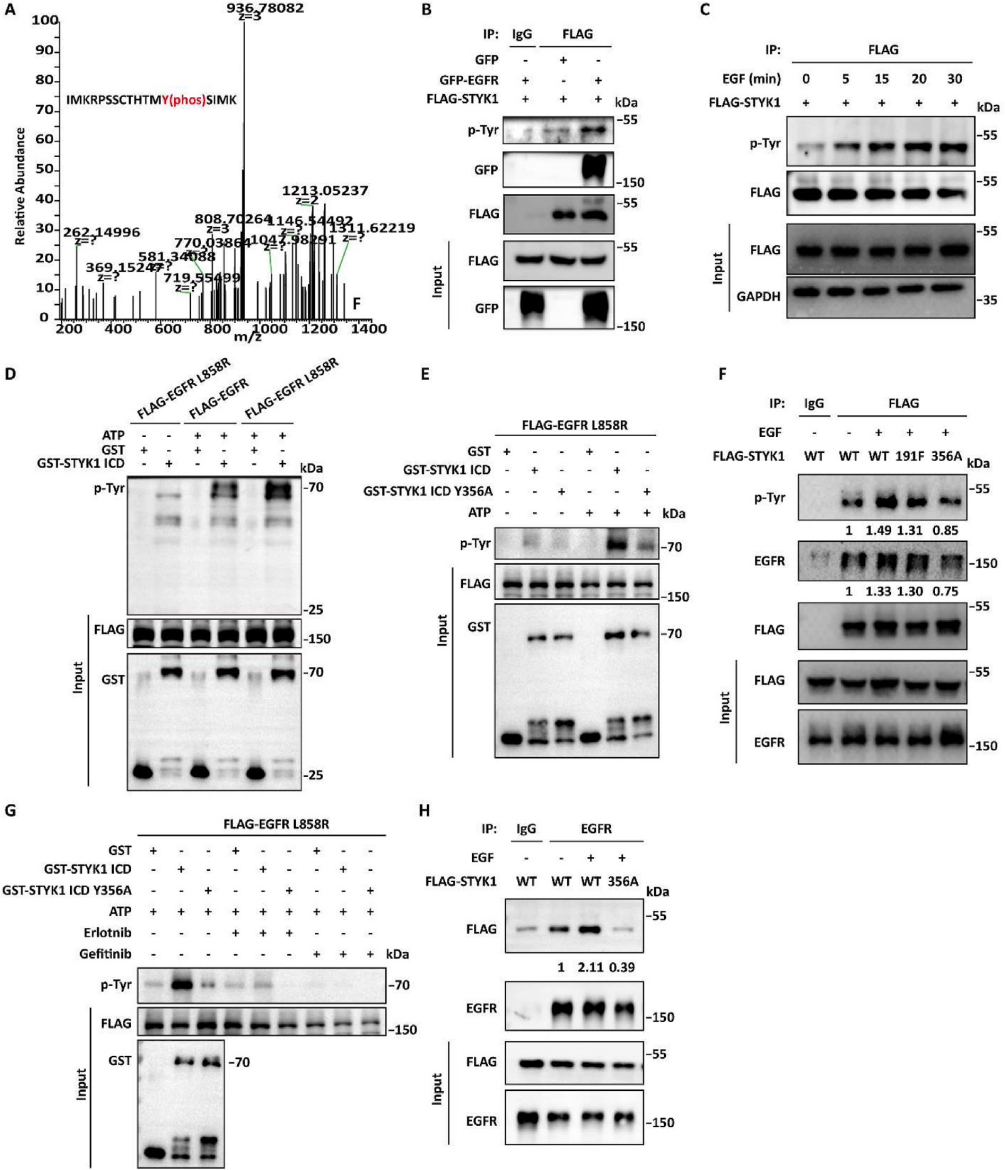
**STYK1 is phosphorylated by EGFR at Y356, which is essential for theSTYK1-EGFRbinding.** (**A**) Mass spectrum showed that STYK1 was phosphorylated at Y356. (**B and C**) Level of total tyrosine phosphorylation of FLAG-STYK1 in HEK293T cells after EGFR transfection or EGF stimulation. Cell lysates were used for IP and western blotting with the indicated antibodies. (**D and E**) FLAG-EGFR or EGFR L858R, obtained from the immunoprecipitate in HEK293T cell lysates using Flag antibody were incubated with the *in vitro* purified GST-STYK1 ICD MT or Y356A protein with or without ATP addition, and then the reaction products were subjected to western blotting. (**F**) Level of total tyrosine phosphorylation of FLAG-STYK1 and STYK1-EGFR interaction in HEK293T cells after EGF stimulation. Cell lysates were used for IP and western blotting with the indicated antibodies. (**G**) Level of total tyrosine phosphorylation of purified GST-STYK1 ICD or Y356A under erlotinib (1 μM) or gefitinib (1 μM) conditions after incubating with FLAG-EGFR L858R obtained from HEK293T cell lysates. (**H**) Interaction between EGFR and STYK1 in HEK293T cells treated with or without EGF. The cell lysates were used for IP and western blotting with the indicated antibodies.

Mass spectrometry data identified that STYK1 was phosphorylated at Y356 (TM**Y**(356)SI) in response to EGF stimulation (Fig. 4A). EGFR increased the level of STYK1 tyrosine phosphorylation compared with that in control cells (Fig. 4B). Moreover, EGF treatment elevated the tyrosine phosphorylation of STYK1 in a time-dependent manner (Fig. 4C). We also confirmed that EGFR could phosphorylate purified GST-STYK1 ICD in the *in vitro* prokaryotic bacterial expression system, and the activated form of EGFR (EGFR L858R) increased much higher of the STYK1 tyrosine phosphorylation level (Fig. 4D).

To test whether EGFR phosphorylates STYK1 at Y356, we replaced Y356 to alanine (A) to generate nonphosphorylatable mutation. We found that STYK1 Y356A significantly reduced STYK1 tyrosine phosphorylation induced by EGFR L858R, whereas Y191F, another phosphorylated tyrosine site by unknown kinase (Zhou et al., 2020a), showed less alteration after EGF stimulation (Fig. 4E and F). Similarly, EGFR L858R mediated STYK1 tyrosine phosphorylation could be significantly inhibited by EGFR-TKI gefitinib or erlotinib (Fig. 4G; Fig. S4A). Moreover, we found reduced binding ability of EGFR with STYK1 after the mutation of Y356A, but not Y191F (Fig. 4F and H). Consistently, deletion of STYK1 291–378 aa (STYK1 Δ291-378), which contains STYK1 tyrosine 356, markedly reduced the interaction between EGFR and STYK1 (Fig. 1G) and inhibited the effects of STYK1 to EGF-induced autophagy regulation (Fig. S2A). These data indicated that STYK1 is phosphorylated by EGFR at Y356, and this phosphorylation of STYK1 is essential for the STYK1-EGFR binding.

### 3.5 EGFR-TKI induced activation of AMPK phosphorylate STYK1 at S304

**Fig. 5:**
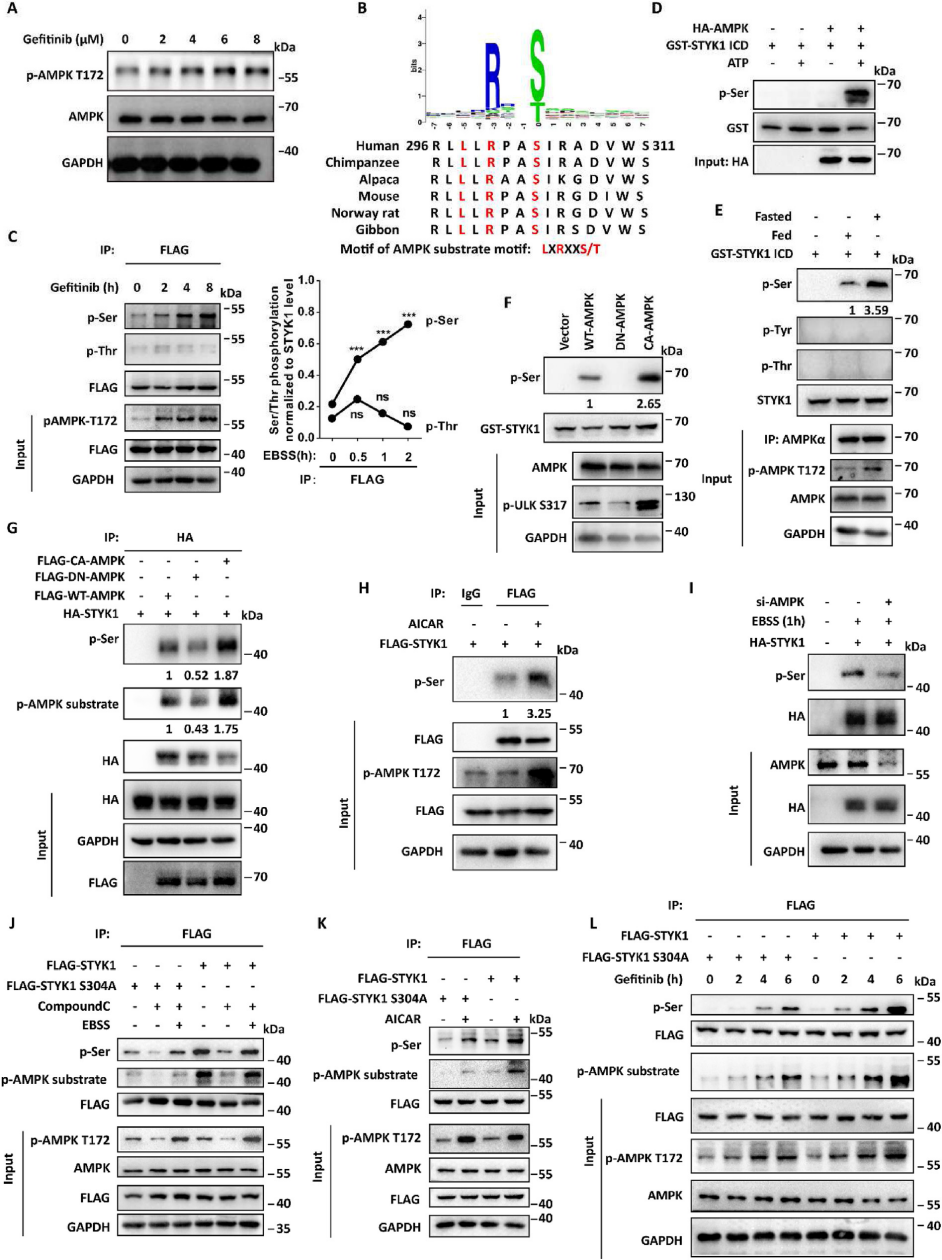
**AMPK phosphorylate STYK1 at S304.** (**A**) Level of p-AMPK T172 in HCC827 cells treated with gefitinib for indicated concentrations. **(B)** Alignment of STYK1 protein sequences from different mammalian species reveals that the sequence surrounding serine 304 in human STYK1 is “LLRPAS(304)IRAD”, which matches the substrate motif of AMPK, LXRXXS/T, and is conserved in higher eukaryotes. **(C)** HCC827 cells expressing FLAG-tagged STYK1 were treated with gefitinib for 0 h, 2 h, 4 h, and 8 h, later the cells were harvest and cell lysates were subjected to immunoprecipitation with an anti-FLAG antibody. Precipitates were then subjected to western blot using the antibody indicated. (**D**) *In vitro* purified GST-STYK1 ICD was incubated with HA-AMPK complex obtained from HEK293T cells with or without ATP. **(E)** The AMPK complexes were obtained from the immunoprecipitate that pre-treated with either fed or fasted HEK293T cell lysates using AMPK antibody. The AMPK complexes were incubated with the *in vitro* purified GST-STYK1 ICD protein, and then the reaction products were subjected to western blot assay. **(F)** The AMPK complexes were obtained from the immunoprecipitate that transfected with either AMPK wild-type, AMPK dominant negative mutant (D157A, AMPK-DN) or AMPK constitutive activated mutant (CA-AMPK) HEK293T cell lysates using AMPK antibody. Then the AMPK complexes were incubated with the *in vitro* purified GST-STYK1 ICD protein at 30 °C for 30 min, and then the reaction products were subjected to western blot assay. **(G)** The phosphorylation level of STYK1 was assessed after transfecting HEK293T cells with the AMPK constitutive activated mutant (CA-AMPK) and the AMPK dominant negative mutant (D157A, AMPK-DN) plasmids. **(H and I)** The effect of AMPK activator AICAR (0.5 mM) or AMPK knockdown to the phosphorylation level of STYK1 serine was assessed in HeLa cells. The cell lysates were immunoprecipitated with anti-FLAG or HA antibody, and precipitates were subjected to western blot assay. (**J-L**) The phosphorylation level of STYK1 and STYK1 S304A mutant were assessed after treatment with or without AMPK inhibitor CompoundC (10 μM), or AMPK activator AICAR, or gefitinib (1 μM). Data were represented as mean ​± ​SD, ∗P ​< ​0.05; ∗∗P ​< ​0.01; ∗∗∗P ​< ​0.001.

Numerous studies have implicated the crosstalk between EGFR and AMPK signaling in EGFR-TKI sensitivity and demonstrated the autophagy induction accompanied with AMPK activation after erlotinib or gefitinib treatment in EGFR mutant NSCLC cells (Chang et al., 2014; Li et al., 2013; Whang et al., 2016; Zhang et al., 2014). We also confirmed that either gefitinib or erlotinib increased the level of pAMPK-T172, which represented elevated AMPK signaling, in time- and does-dependent manner in HCC827 cells (Fig. 5A; Figs. S5A and B). We observed one conserved sequence surrounding serine 304 in human STYK1 “LLRPAS(304)”, which matches the optimal AMPK substrate motif LXRXXS/T (Fig. 5B), we then asked whether EGFR-TKI induced AMPK activation could phosphorylate STYK1 thus enhance autophagy activity. To this end, we first examined the level of serine phosphorylated STYK1 after gefitinib treatment. The results showed that gefitinib time-dependently increased serine phosphorylated STYK1 level, but not threonine phosphorylated level (Fig. 5C). Using the *in vitro* kinase assay, we showed that the phosphorylation level of STYK1 serine was improved after incubation the prokaryotic expressed GST-STYK1 ICD with the AMPK immunoprecipitate from AMPK transfected or fasted HEK293T cell lysate (Fig. 5D and E). Moreover, we confirmed that the level of serine phosphorylated STYK1 was much higher while purified GST-STYK1 ICD incubated with continuously activated AMPK (active-AMPK) immunoprecipitate compared with that in normal AMPK immunoprecipitate incubating product. In contrast, dominant negative (D157A) form of AMPK (AMPK-DN) showed no effects on STYK1 serine phosphorylation (Fig. 5F). Furthermore, we found that the level of STYK1 serine phosphorylation was much more enhanced in cells transfected with active-AMPK and reduced in AMPK-DN transfected cells compared with wild-type AMPK (Fig. 5G). Consistently, the phosphorylation of STYK1 serine also increased after treatment with AICAR, an AMPK activator (Fig. 5H) and down-regulated in AMPKɑ-deleted fasted HeLa cells (Fig. 5I).

To investigate whether AMPK phosphorylates STYK1 at S304, STYK1 S304A mutant was generated and transfected in HEK293T cells. The results showed that STYK1 S304A mutant significantly reduced the level of STYK1 serine phosphorylation in the treatment with or without AMPK inhibitor CompoundC, or AMPK activator AICAR (Fig. 5J-L). Moreover, we confirmed that STYK1 S304A mutant markedly reduced gefitinib induced STYK1 serine phosphorylation (Fig. 5L). Additionally, we also tested the effect of STYK1 S304 to autophagy activity in A549 and HCC827 cells, and the results revealed that the activity of autophagy was much more decreased in cells transfected with S304A, but increased in cells transfected with S304D, compared with wide-type STYK1 in the basal condition represented by the level of LC3-II accumulation (Fig. S5C). These results indicated that EGFR-TKI induced AMPK activation phosphorylates STYK1 at serine 304 thus enhances autophagy activity.

### 3.6 Phosphorylation of STYK1 S304 collaborated with Y356 to enhance autophagy activation upon EGFR-TKI treatment

**Fig. 6:**
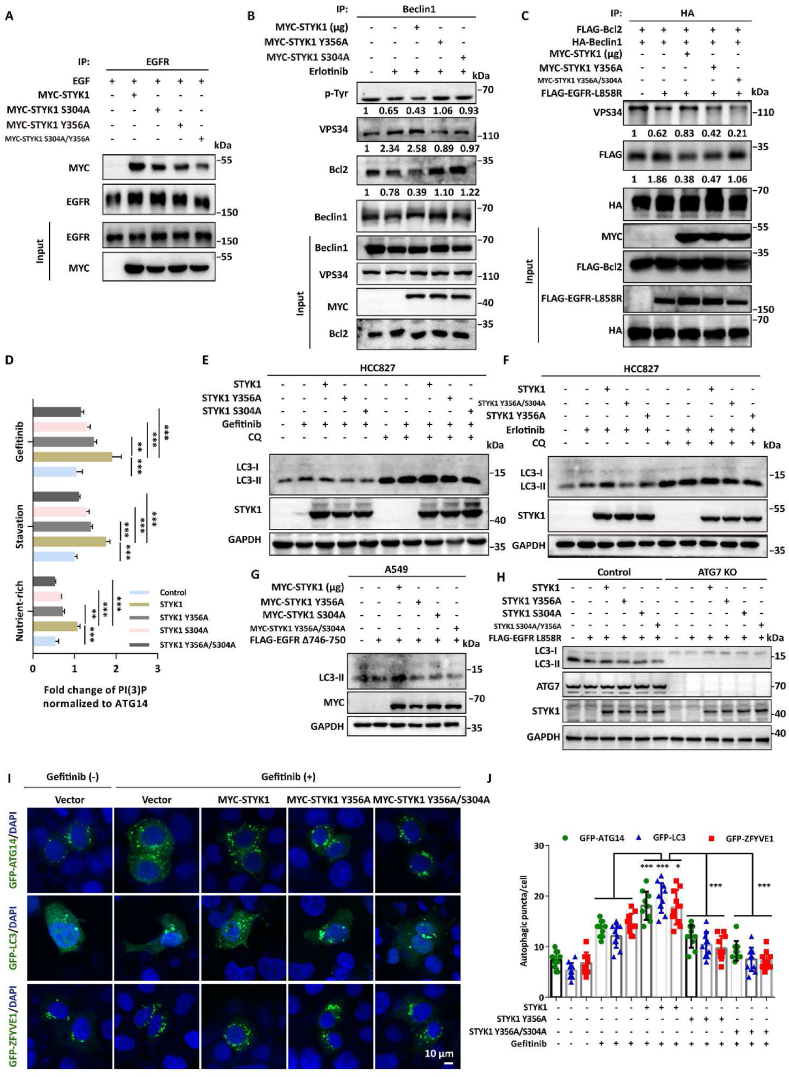
**Phosphorylation of STYK1 S304 collaborated with Y356 to enhance autophagy activation.** (**A**) Interaction between EGFR and STYK1 or its mutants as indicated in HEK293T cells treated with EGF. Cell lysates were used for IP and western blotting with the indicated antibodies. (**B**) HCC827 cells transfected with either MYC-STYK1 or its mutants Y356A/S304A expression plasmid for 48 h treated with erlotinib (1 μM) were harvest and cell lysates were subjected to co-IP and western blotting with the indicated antibodies. (**C**) The assembly of Beclin1 interactomes after STYK1 or its mutants overexpressing as indicated in HEK293T cells transfected with EGFR L858R. Cell lysates were used for IP and western blotting with the indicated antibodies. (**D**) Different PtdIns3K-C1 complex components from the control or STYK1 overexpressed HCC827 cells under starvation or gefitinib (1 μM) treatment conditions were immunoprecipitated by the ATG14 antibody. PIK3C3 activity was measured by analyzing PtdIns3P production using the ELISA assay described in the Materials and Methods. The PtdIns3P fold change was calculated based on the concentration of PtdIns3P and was normalized to the amount of ATG14 used in the assay. (**E and F**) Western blotting analysis were used to analyze the protein expression levels of LC3-II in HCC827 cells overexpressing MYC-STYK1 and its mutants with or without gefitinib (1 μM), erlotinib (1 μM) and CQ (10 μM) treatment. (**G**) Western blotting analysis were used to analyze the protein expression levels of LC3-II in A549 cells co-expressing EGFR Δ746-750 and MYC-STYK1 mutants. (**H**) Western blotting analysis were used to analyze the protein expression levels of LC3-II in MEF cells with ATG7 knockout after EGFR Δ746-750 and MYC-STYK1 mutants co-expressing. (**I and J**) Representative confocal images of GFP-ATG14, GFP-LC3 and GFP-ZFYVE1 distribution in MYC-STYK1 and its mutants expressing HCC827 cells treated with or without gefitinib (1 μM). The number of GFP-ATG14, GFP-LC3 and GFP-ZFYVE1 puncta was quantified (n ​= ​10). Scale bars, 10 ​μm. Data were represented as mean ​± ​SD, ∗P ​< ​0.05; ∗∗P ​< ​0.01; ∗∗∗P ​< ​0.001.

As the site of STYK1 serine 304 is closed to tyrosine 356 and deletion of STYK1 291–378 aa significantly decreased the interaction between STYK1 and EGFR (Fig. 1G), we asked whether STYK1 S304 phosphorylation have effects on the STYK1-EGFR binding ability. STYK1 S304A mutant was then transfected to HEK293T cells and we found that the amount of EGFR was significantly decreased in the immunoprecipitate of STYK1 S304A and S304/Y356 double mutant compared with that in wild-type STYK1 (Fig. 6A; Fig. S6A). Both STYK1 S304A and Y356A mutants markedly increased the level of Beclin1 tyrosine phosphorylation compared with wild-type STYK1 upon erlotinib treatment in HCC827 cells (Fig. 6B). Consistently, STYK1 S304A or Y356A inhibited the dimerization of Beclin1 compared with the effects of wild-type STYK1 (Fig. S6B). Moreover, STYK1 S304A and Y356A mutants decreased the interaction between Beclin1 and VPS34, and increased the interaction between Beclin1 and Bcl2, respectively (Fig. 6B). Double mutations of STYK1 (STYK1 Y356A/S304A, hereafter refers to as “STYK1 DM”) showed aggravated effects on the Beclin1-VPS34 and Beclin1-Bcl2 interactions compared with STYK1 Y356A alone in HEK293T cells transfected with EGFR L858R (Fig. 6C). We next analyzed whether the state of STYK1 phosphorylation affects the ATG14-linked VPS34 kinase activity. The *in vitro* kinase assay revealed that STYK1 Y356A or S304A significantly decreased the ATG14-linked VPS34 activity under the control, starvation or gefitinib treatment condition compared with that in wild-type STYK1 transfected HCC827 cells. STYK1 DM mutant showed much less VPS34 kinase activity under these conditions (Fig. 6D). Moreover, STYK1 S304A significantly inhibited the assembly of PtdIns3K-C1 complex, whereas S304D showed enhanced effects (Fig. S6C).

To investigate the role of STYK1 phosphorylation in the regulation of EGF-induced autophagy inhibition, we firstly tested the level of LC3-II in the STYK1 Y356A or S304A transfected NSCLC cells. The results revealed that either STYK1 Y356A or STYK1 S304A mutant inhibited autophagy flux in HCC827 cells under gefitinib treatment condition compared with wide-type STYK1 (Fig. 6E). Moreover, STYK1 DM mutant showed much less autophagy flux compared with that in cells transfected with STYK1 Y356A alone (Fig. 6F). Consistently, similar effects were found in A549 cells transfected with EGFR Δ746-750 active form (Fig. 6G). ATG7 knockout completely abolished the effects of STYK1 and its mutants on autophagy under EGFR L858R transfected condition in MEF cells (Fig. 6H). Furthermore, either STYK1 Y356A, S304A or STYK1 DM mutant decreased the formation of autophagic puncta after gefitinib treatment, as indicated by GFP-ATG14, GFP-WIPI1 and GFP-LC3 (Fig. 6I). These results indicated that phosphorylation of STYK1 S304 is also required for the interaction between EGFR and STYK1, and collaborated with Y356 to enhance autophagy activation upon EGFR-TKI treatment.

### 3.7 STYK1 depletion increases the sensitivity of NSCLC cells to EGFR-TKI *in vitro* and *in vivo*

**Fig. 7:**
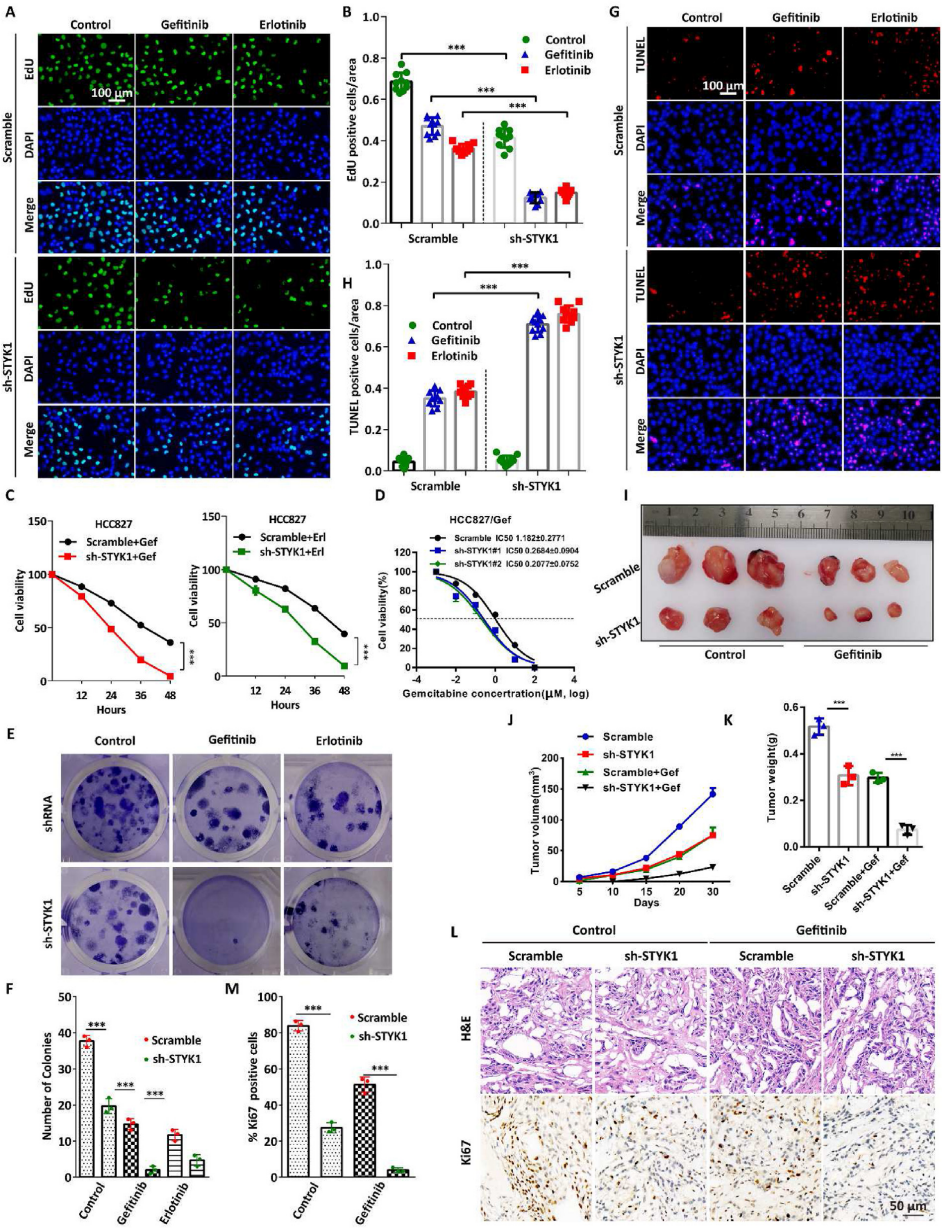
**STYK1 depletion increases the sensitivity of NSCLC cells toEGFR-TKI*****in vitro*****and*****in vivo.*** (**A and B**) DNA synthesis ability of the cells stably expressing STYK1 shRNA were assessed by EdU assays under gefitinib (0.5 μM, 12 h) or erlotinib (0.5 μM, 12 h) treatment conditions. The number of EdU-positive cells was quantified. Scale bars: 100 μm. (**C**) MTT assays were performed to examine the effects of STYK1 knockdown on cell viability. (**D**) HCC827 cells with stable STYK1 knockdown and were treated with gefitinib at different concentrations for 48 h, and cell viability was then measured by MTT assay. (**E and F**) Colony formation assays were performed to assess the effects of STYK1 knockdown on the proliferation of HCC827 cells treated with or without gefitinib or erlotinib treatment. (**G and H**) Apoptotic cells among STYK1 knockdown HCC827 cells treated with gefitinib or erlotinib were analyzed by TUNEL assay, the number of TUNEL-positive cells was quantified. Scale bars: 100 μm. (**I**) Excised tumors in different groups were shown. (**J**) Growth curves showing the changes in the tumor volume in mice in different groups every 5 days from the injection. (**K**) Weight of the excised tumors in each group. (**L and M**) Representative H&E staining images and immunohistochemical images of Ki67 in excised tumors tissues. Data were represented as mean ​± ​SD, ∗P ​< ​0.05; ∗∗P ​< ​0.01; ∗∗∗P ​< ​0.001.

To determine the role of STYK1 in the EGFR-TKI sensitivity of NSCLC, we firstly stably overexpressed STYK1 shRNA in HCC827 cells which contains the Δ746-750 EGFR activating mutation using lentiviral transfection. EdU incorporation assays were carried out to assess the changes in DNA synthesis in HCC827 cells exposed with gefitinib or erlotinib. The results showed that STYK1 depletion significantly decreased EdU positive cells in either gefitinib or erlotinib treatment conditions (Fig. 7A and B). Cell viability assay showed that the depletion of STYK1 increased the sensitivity of HCC827 cells to gefitinib and erlotinib compared with that of the control cells (Fig. 7C). Moreover, cells were also exposed with indicated concentrations of gefitinib or erlotinib for 48 h and cell viability assay showed that the depletion of STYK1 increased the sensitivity of HCC827 cells to either gefitinib or erlotinib and decreased the IC50 value compared with that of the control cells (Fig. 7D; Fig. S7A). Additionally, we found that STYK1 Y356A, S304A mutants or double-mutant significantly decreased EdU positive cells compared with wide-type overexpressed group in STYK1-deficient cells (Figs. S7A and B). Consistently, STYK1 depletion attenuated HCC827 cell colony formation activities (Fig. 7E and F). Moreover, we investigated whether STYK1 affects the apoptosis rate of HCC827 cells induced by gefitinib or erlotinib. To that end, TUNEL assay was performed and the results showed that STYK1 depletion significantly increased the number of TUNEL-positive cells compared with that among the control cells (Fig. 7G and H).

To further assess the impact of STYK1 on NSCLC cell sensitivity to EGFR-TKI *in vivo*, BALB/c nude mice bearing subcutaneous NSCLC xenograft tumors derived from HCC827 cells stably expressing STYK1 shRNA were treated with 25 mg/kg gefitinib. After 4 weeks treatment, we found that the growth of the tumors in the group with STYK1 depletion were much slower than that in the control group with or without gefitinib treatment (Fig. 7I). And the tumor volume and weight were consistent smaller (Fig. 7J and K). Moreover, STYK1 depletion also contributed to the downregulation of Ki67 in xenograft tumor tissues (Fig. 7L and M). Taken together, these data indicate that STYK1 depletion increases the sensitivity of NSCLC to EGFR-TKI *in vitro* and *in vivo*.

## 4 Discussion

Increased researches have implicated that autophagy contributes to the resistance of EGFR-TKIs and thus affects the therapeutic effect (Chen et al., 2021; Kwon et al., 2019). Clinical trials have also been conducted using autophagy inhibitors in combination with EGFR-TKIs for the treatment of NSCLC (Liu et al., 2015). EGFR was previously reported to inhibit autophagy independent of mTOR but involve in the interaction between EGFR and Beclin1 (Wei et al., 2013). However, the cross-talk between EGFR and other interacting partners and the molecular mechanisms in autophagy regulation and TKIs resistance remains obscure. We have recently reported that STYK1 functions as a positive regulator of autophagy through directly binding to the Beclin1 and ATG14, thus activating the assembly and the kinase activity of PtdIns3K-C1 complex in basal state (Zhou et al., 2020a). In the present study, through the interactome screening of STYK1 using mass spectrometry assay, we demonstrated the new roles of STYK1 that interacts with EGFR under EGF stimulation and reverses its effects to autophagy activity through altering EGFR mediated Beclin1 phosphorylation and Beclin1 interactome assembly. Moreover, we also confirmed the positive role of STYK1 in EGFR-TKIs resistance of NSCLC patients harboring EGFR active mutant.

There are several other classifications of resistance mechanisms for EGFR-TKIs that have been validated in patients besides the secondary mutation of EGFR (EGFR T790M). Studies have highlighted that acquired resistance to EGFR-TKIs converge into EGFR downstream signaling transductions, including the RAS/RAF/MEK/ERK and PI3K/Akt/mTOR (Koustas et al., 2017). Our previous study have also indicated that STYK1 enhances the mTOR activity according to the increased phosphorylation of downstream effectors EIF4EBP1 (Wang et al., 2016), which is consistent with previous reports that STYK1 promote MAPK and PI3K/ATK signaling in the upstream of the mTOR pathway (Broix et al., 2016). Although it is contradictory that mTOR plays negative roles in autophagy (Hosokawa et al., 2009), it shows the collaborate of mTOR and autophagy mediated by STYK1 at least in the cases of EGFR-TKIs resistance or pancreatic cancer with activating KRAS mutations and high basal autophagy (Rosenfeldt et al., 2013). Moreover, pathways intrinsic and extrinsic stresses, including iatrogenic stress, trigger robust EGFR trafficking and signaling to provide cancer cells with a survival benefit and resistance to therapeutics (Tan et al., 2016). Autophagy was reported to facilitate EGFR recycling and signaling and autophagy share multiple commonalities with the endocytic pathway such as phosphoinositide-3-phosphate (PtdIns3P) (Fraser et al., 2017, 2019; Kutateladze, 2010). We have been figured out that STYK1 plays an essential role in PtdIns3P generation (Zhou et al., 2020a). In the present study, we also showed that STYK1 knockdown decreased the total level of EGFR, suggesting roles of STYK1 in EGFR recycling, stability and downstream activity (Fig. 1I).

EGFR signaling regulates global metabolic pathways in EGFR-mutated lung adenocarcinoma and EGFR-TKIs such as erlotinib and gefitinib are reported to drive energetic stress thus activate the AMPK pathway in EGFR (del19) lung tumors (Makinoshima et al., 2014; Momcilovic et al., 2017; Whang et al., 2016). In the present study, we also confirmed that either gefitinib or erlotinib elevated AMPK signaling in both time- and does-dependent manner (Fig. 5A). Moreover, we further investigated that elevated AMPK activity could phosphorylate STYK1 at serine 304, which showed positive effects on gefitinib or erlotinib induced autophagy. Furthermore, STYK1 S304A mutant showed reduced xenografts with gefitinib treatment. Consistently with the notion that dual inhibition of EGFR and AMPK signaling in HCC827 xenografts led to metabolic crisis and eventually tumor cell death (Chang et al., 2015; Momcilovic et al., 2017). Additionally, we also noticed that the level of STYK1 serine phosphorylation was reduced even when STYK1 S304 mutated to alanine, suggesting another AMPK phosphorylation site(s) in STYK1 protein. From the human protein reference database, we predicted another serine site that could be phosphorylated by AMPK, which matches the AMPK substrate motif [M/V/L/I/F][R/K/H]XX[pS/pT]XXX[M/V/L/I/F] at STYK1 S91(Amanchy et al., 2007). It is worth to confirm the relationship between STYK1 S91 and AMPK, and its role in EGFR-TKI resistance.

**Fig. 8:**
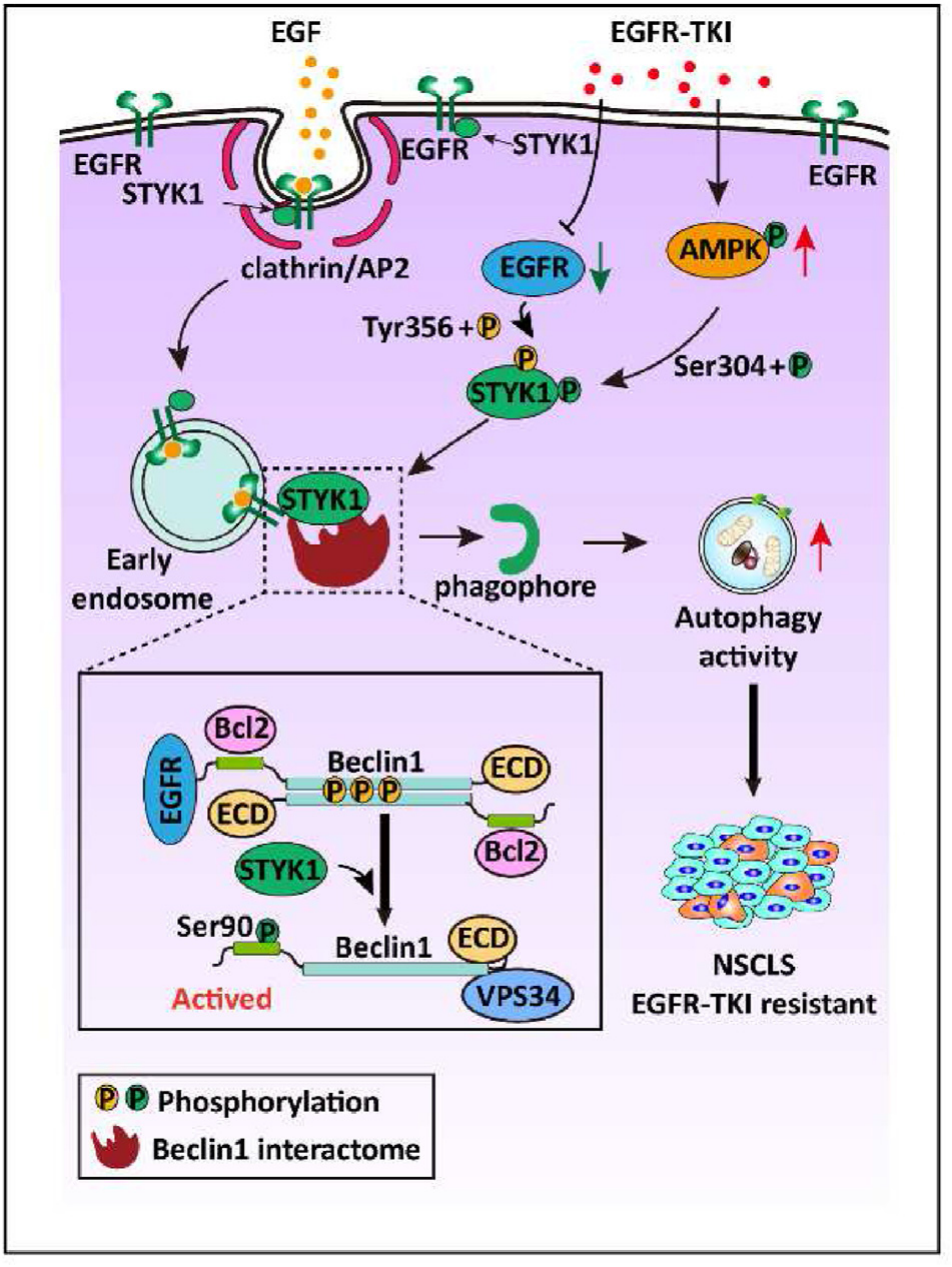
**Schematic illustration depicting a proposed model of the molecular mechanism of thecross-talkbetween STYK1 and EGFR in autophagy regulation andEGFR-TKIresistance in NSCLC.**

Collectively, in the present study, we demonstrated that STYK1 interacts with EGFR and regulates autophagy flux on epidermal growth factor stimulation or EGFR-TKIs therapy. We also indicated that tyrosine 356 phosphorylated STYK1 binds more EGFR to enhance autophagy thus promoting EGFR-TKIs resistance compared with wild-type STYK1. Moreover, EGFR-TKIs induced AMPK activation amplifies the effects of STYK1 on EGFR induced autophagy inhibition (Fig. 8). Additionally, our study detailed roles of STYK1 in the regulation of autophagy which showed interplay with EGFR-TKI and also provided a proof of concept for a therapeutic strategy against NSCLC development and EGFR-TKI resistance.

## Author contributions

Jingfeng Tang and Cefan Zhou: Conceptualization, Methodology, Software. Cefan Zhou, Xueying Dong, Xuehong Qian, Miao Hu, Kai Liang, Yanyan Liang, Rui Zhang, Yuan Huang, Hao Lyu and Shuai Xiao: Data curation. Cefan Zhou: Writing- Original draft preparation. Cefan Zhou, Ming Wang and Yongfei Tang: Visualization, Investigation. Jingfeng Tang: Supervision. Declan William Ali, Marek Michalak Xing-Zhen Chen and Jingfeng Tang: Writing- Reviewing and Editing.

## Funding

This work was supported by the 10.13039/501100001809National Natural Science Foundation of China (32070726 to J.F.T., 31871176 to X.Z.C.), Wuhan Science and Technology Project (2019020701011475 to J.F.T), National Natural Science Foundation of Hubei (2020CFA073 to J.F.T.), Doctoral Start-up Foundation of 10.13039/501100002948Hubei University of Technology (BSQD2020035 to C.F.Z).

## Availability of data and materials

All data generated or analyzed during this study are included within the article.

## Ethics approval and consent to participate

The experimental protocol for animal studies was reviewed and approved by the institutional animal care and use committee of Hubei University of Technology.

## Consent for publication

All authors agreed on the manuscript.

## Declaration of competing interest

The authors declare no conflict of interest.
