# Supplementary material for: Phosphorylated STYK1 restrains the inhibitory role of EGFR in autophagy initiation and EGFR-TKIs sensitivity
Source: Cell Insight. 2022 Jun 16;1(4):100045. doi: 10.1016/j.cellin.2022.100045 (PMC10120315; doi:10.1016/j.cellin.2022.100045)
Supplement: Multimedia component 1 [file mmc1.docx]

# Supplementary Figures and Figure legends


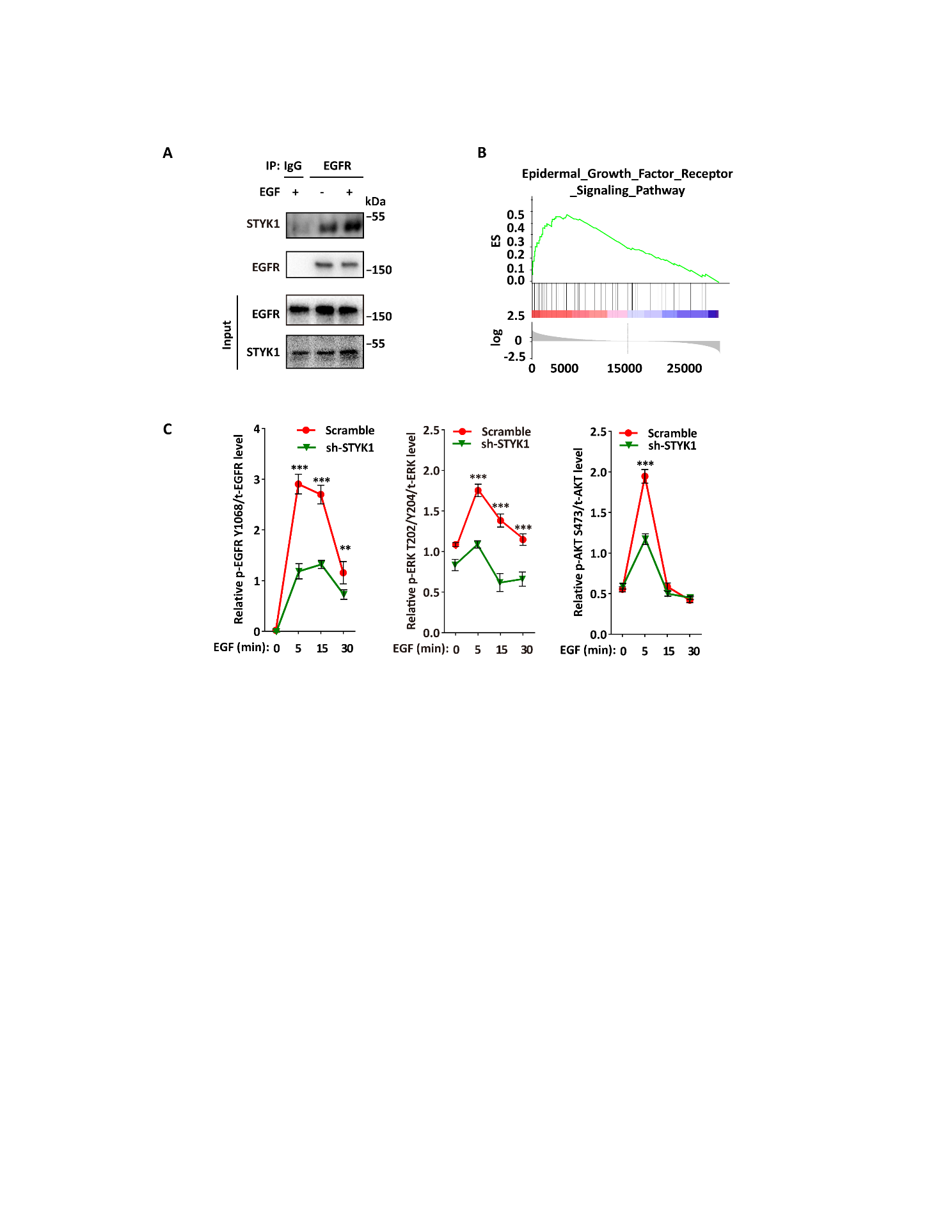


**Figure S1. STYK1 promotes EGFR signaling, related to Figure 1**.

**(A)** The interaction between endogenous STYK1 and EGFR after treatment with EGF for 15 min. (**B**) Gene set enrichment analysis (GSEA) using the TCGA lung cancer database revealed many genes that correlated with EGFR signaling were enriched in lung cancer patients with high STYK1 expression. (**C**) Quantification of the protein expression levels of p-EGFR Y1068, p-ERK1/2 T202/Y204 and p-AKT S473 in A549 cells stably knockdown STYK1 upon treatment with EGF for indicated times. Data were represented as mean ± SD, *P <0.05; **P < 0.01; ***P < 0.001.


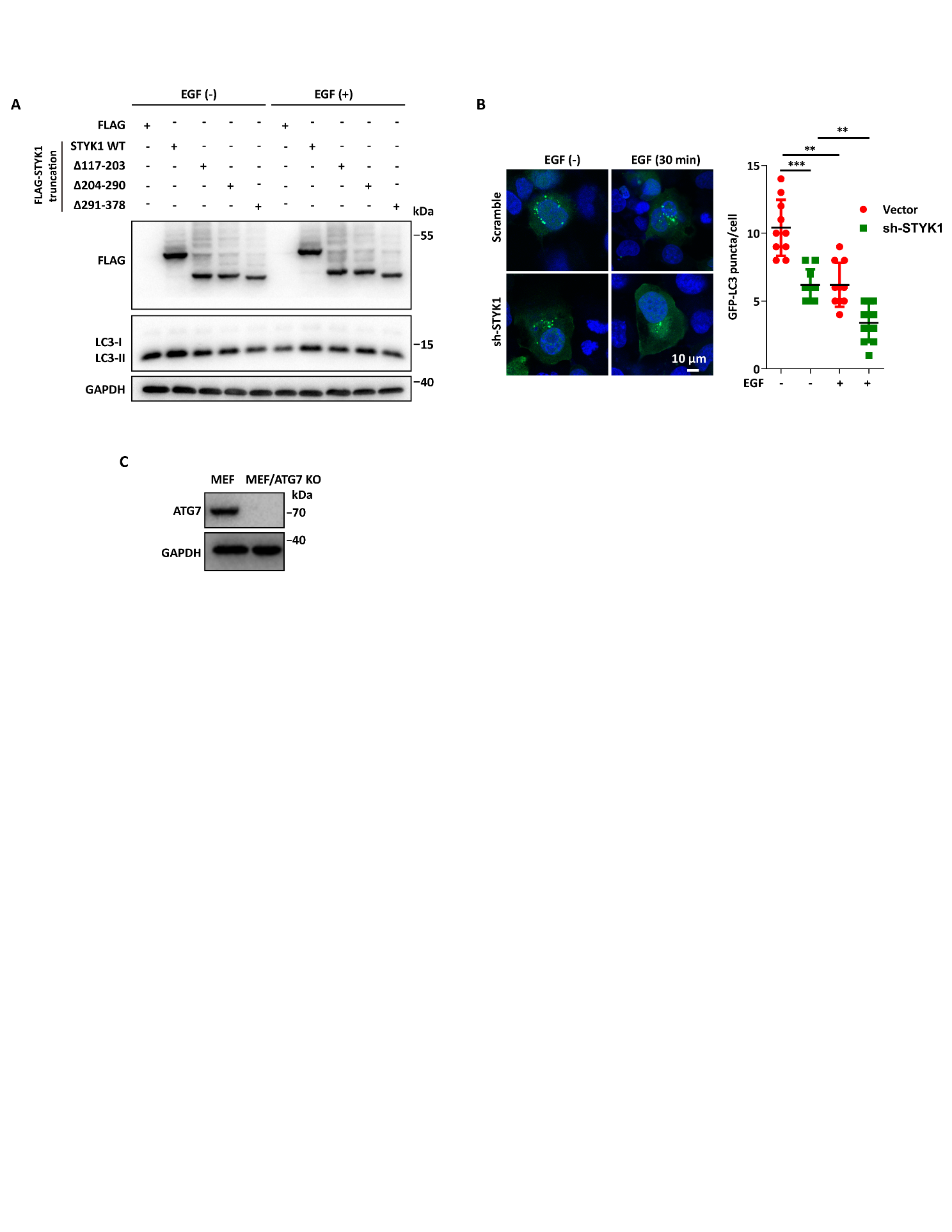


**Figure S2.** **STYK1 inhibits the effects of EGFR to autophagy, related to Figure 2.**

(**A**) Western blotting analysis were used to analyze the protein expression levels of LC3-II in A549 cells overexpressing FLAG-STYK1 and its mutants with or without EGF treatment. (**B**) Representative confocal images of GFP-LC3 distribution in STYK1 knockdown HeLa cells with or without EGF treatment. The number of GFP-LC3 puncta was quantified (n =10). Scale bars, 10 µm. (**C**) Western blotting analysis were used to analyze the protein expression levels of ATG7 in MEF cells.


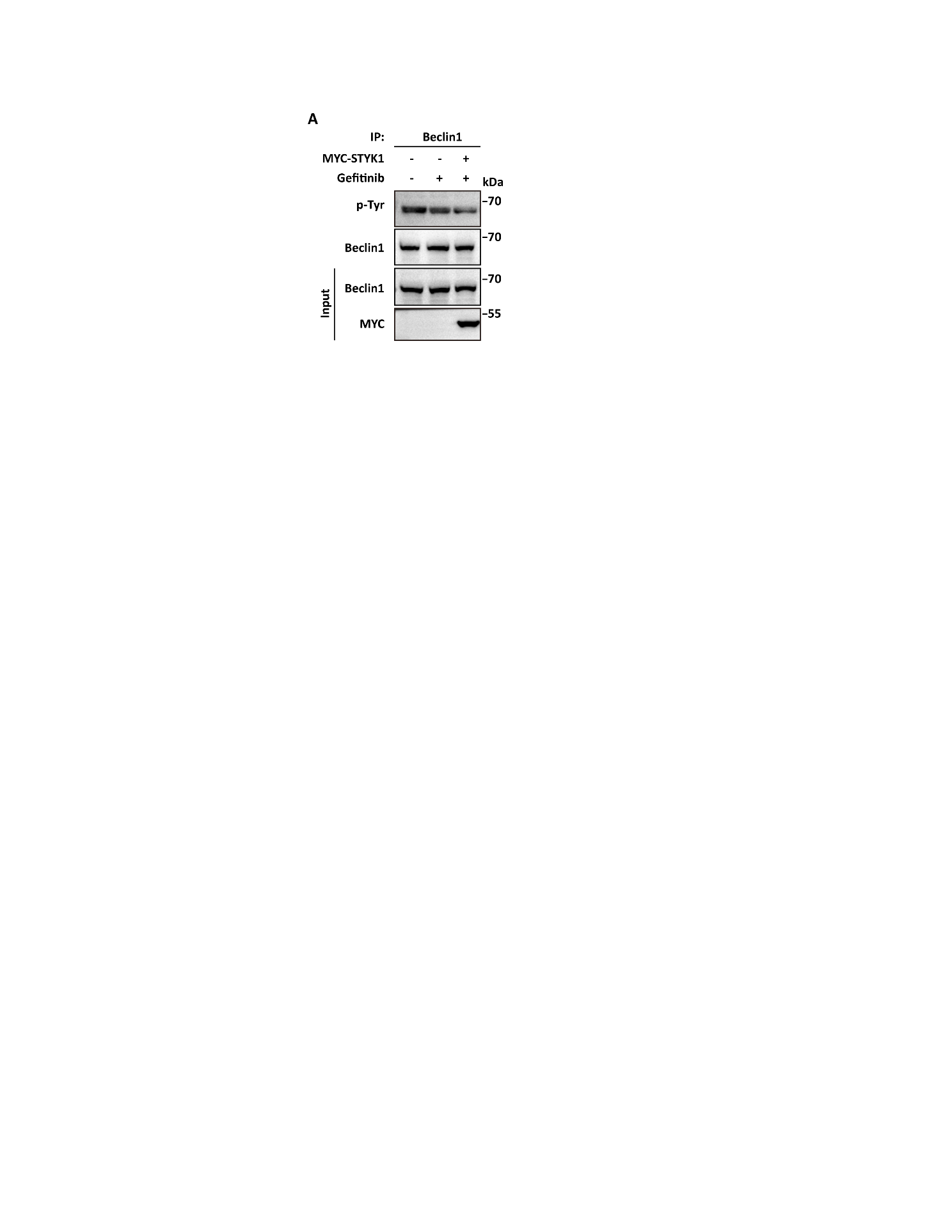


**Figure S3.** **STYK1 inhibits Beclin1 tyrosine phosphorylation, related to Figure 3.**

The level of total tyrosine phosphorylation of Beclin1 after STYK1 overexpression in HCC827 cells treated with gefitinib. The cell lysates were used for IP and western blotting with the indicated antibodies.

**
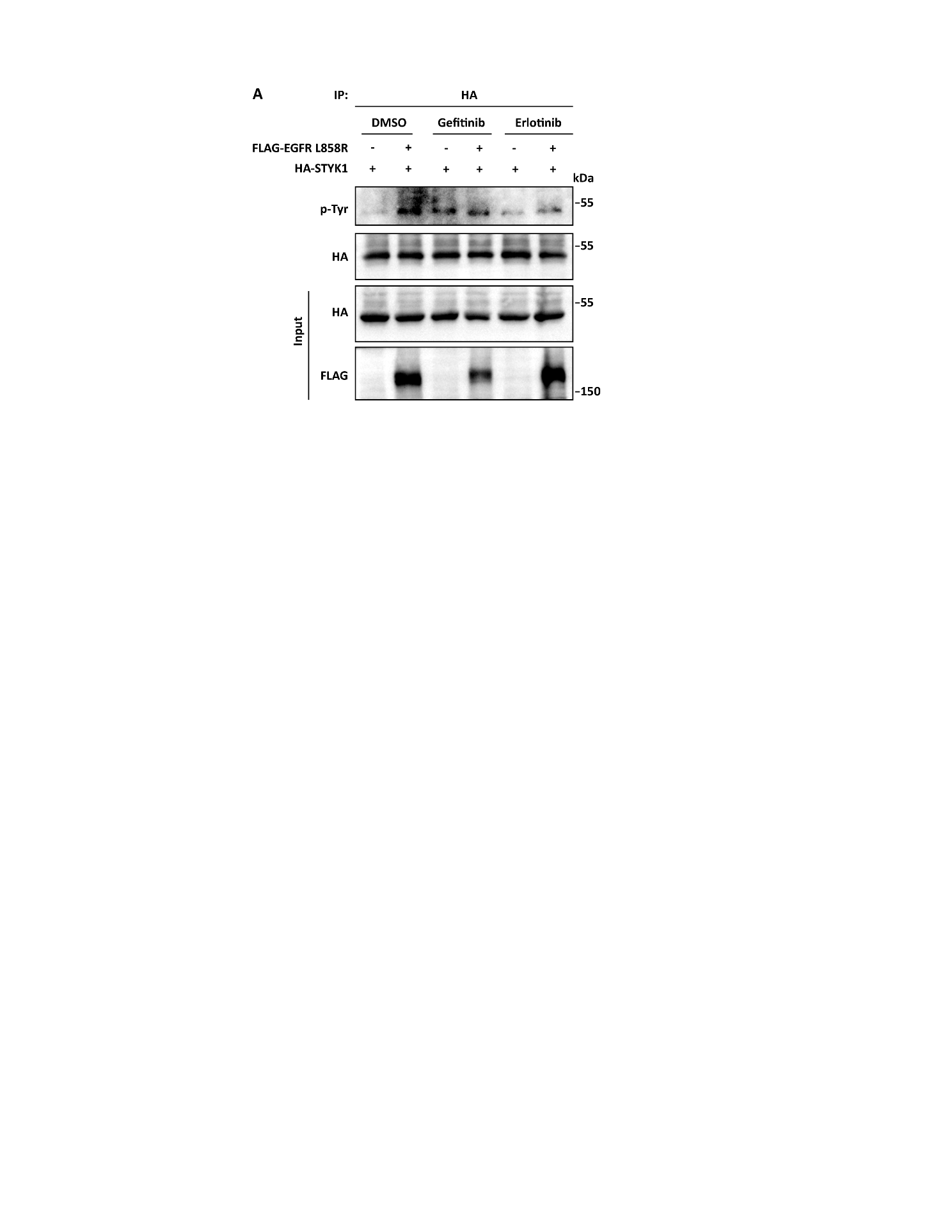
**

**Figure S4. EGFR binds STYK1 in a kinase dependent manner, related to Figure 4.**

The interaction between EGFR L858R and STYK1 in HEK293T cells treated with or without gefitinib and erlotinib. The cell lysates were used for IP and western blotting with the indicated antibodies.


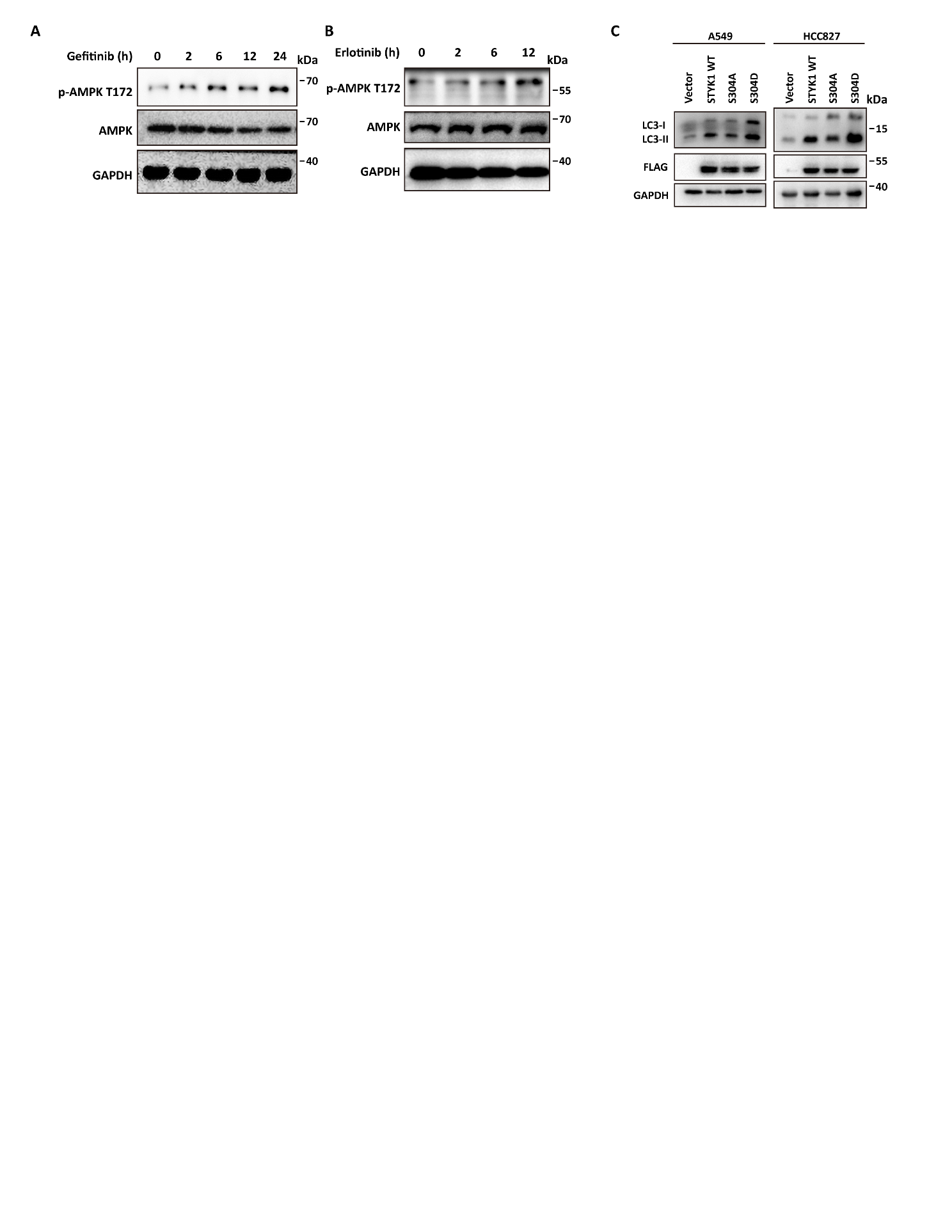


**Figure S5. STYK1 S304 phosphorylation promotes autophagy, related to Figure 5.**

**(A and B)** The level of p-AMPK T172 in HCC827 cells treated with gefitinib for indicated times. (**C**) Western blotting analysis were used to analyze the protein expression levels of LC3-II in A549 and HCC827 cells overexpressing FLAG-STYK1 and its mutants S304A or S304D.


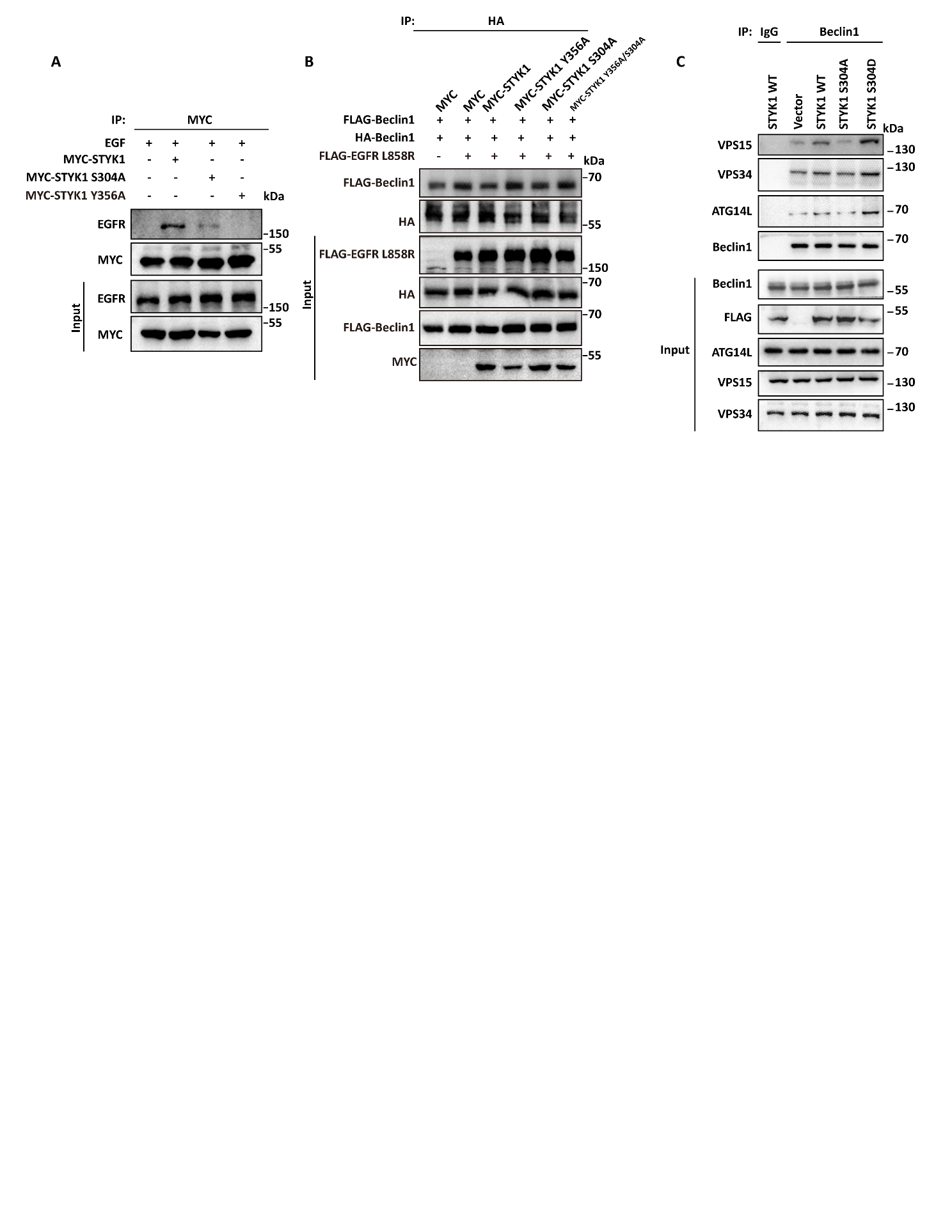


**Figure S6.** **STYK1 S304 collaborated with Y356 to enhance autophagy activation, related to Figure 6.**

(**A**) The interaction between EGFR and STYK1 or its mutants as indicated in HEK293T cells treated with EGF. The cell lysates were used for IP and western blotting with the indicated antibodies. (**B**) The level of Beclin1 dimerization after STYK1 and its mutants overexpression in EGFR L858R transfected HEK293T cells. The cell lysates were used for IP and western blotting with the indicated antibodies. (**C**) HCC827 cells were transfected with wild-type STYK1 and its mutants for 48 h, then cell lysates were subjected to IP with Beclin1 antibodies. Precipitates were then subjected to western blotting analysis. Empty vector or rabbit IgG was used as the control.


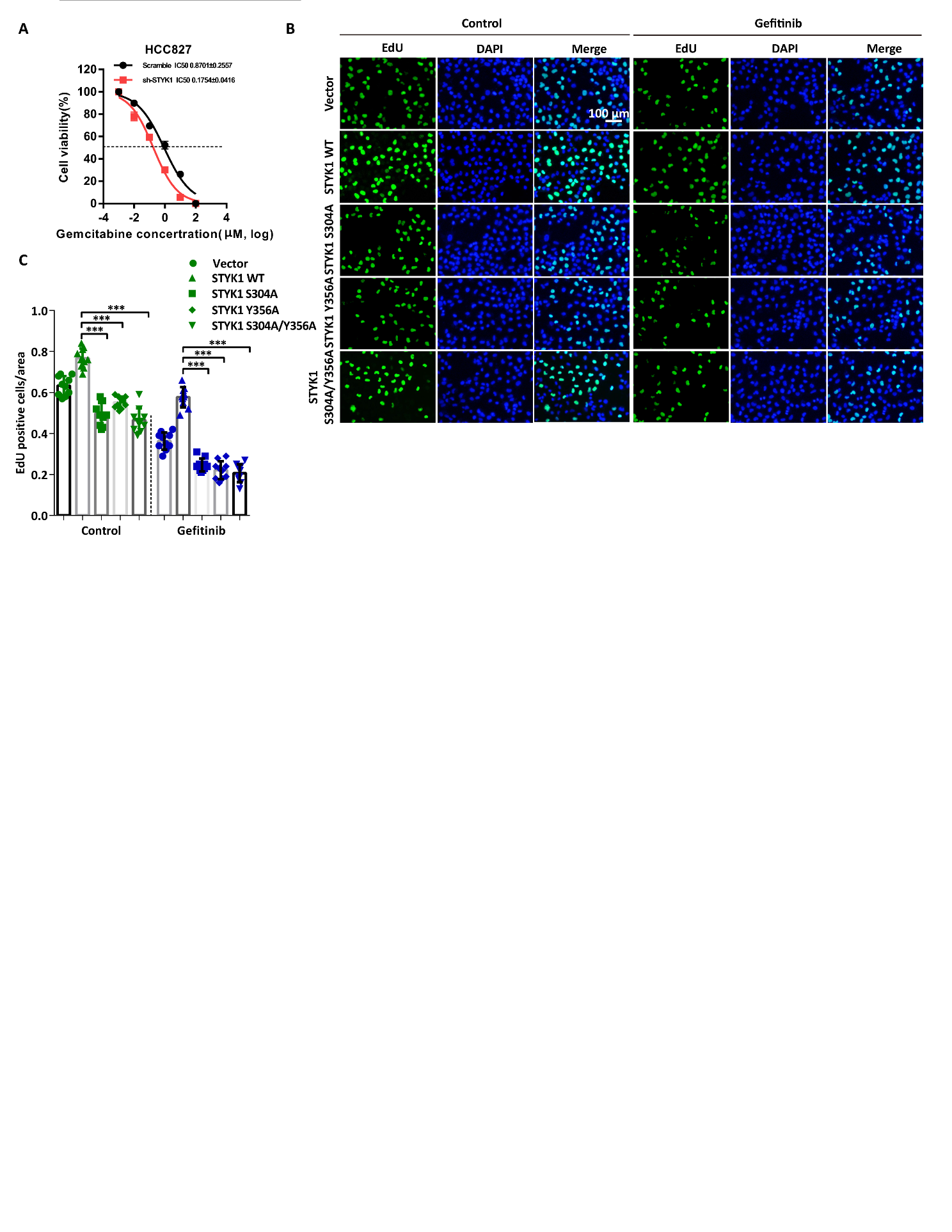


**Figure S7. STYK1 knockdown increases the sensitivity of NSCLC cells to erlotinib, related to Figure 7.**

(**A**) HCC827 cells with stable STYK1 knockdown and were treated with gefitinib at different concentrations for 48 h, and cell viability was then measured by MTT assay. (**B and C**) DNA synthesis ability of the cells stably expressing wild-type STYK1, STYK1 S304A, STYK1 Y356A or STYK1 S304A/Y356A double mutant were assessed by EdU assays under gefitinib (0.5 μM, 12 h) treatment conditions. The number of EdU-positive cells was quantified. Scale bars: 100 μm.
